# Genome-wide introgression among distantly related *Heliconius* butterfly species

**DOI:** 10.1186/s13059-016-0889-0

**Published:** 2016-02-27

**Authors:** Wei Zhang, Kanchon K. Dasmahapatra, James Mallet, Gilson R. P. Moreira, Marcus R. Kronforst

**Affiliations:** Department of Ecology & Evolution, University of Chicago, Chicago, IL 60637 USA; Department of Biology, University of York, York, YO10 5DD UK; Department of Organismic & Evolutionary Biology, Harvard University, Cambridge, MA 02138 USA; Departamento de Zoologia, Universidade Federal do Rio Grande do Sul, Porto Alegre, RS 91501-970 Brazil

**Keywords:** Adaptation, Gene flow, Introgression, Mimicry

## Abstract

**Background:**

Although hybridization is thought to be relatively rare in animals, the raw genetic material introduced via introgression may play an important role in fueling adaptation and adaptive radiation. The butterfly genus *Heliconius* is an excellent system to study hybridization and introgression but most studies have focused on closely related species such as *H. cydno* and *H. melpomene*. Here we characterize genome-wide patterns of introgression between *H. besckei*, the only species with a red and yellow banded ‘postman’ wing pattern in the tiger-striped silvaniform clade, and co-mimetic *H. melpomene nanna*.

**Results:**

We find a pronounced signature of putative introgression from *H. melpomene* into *H. besckei* in the genomic region upstream of the gene *optix*, known to control red wing patterning, suggesting adaptive introgression of wing pattern mimicry between these two distantly related species. At least 39 additional genomic regions show signals of introgression as strong or stronger than this mimicry locus. Gene flow has been on-going, with evidence of gene exchange at multiple time points, and bidirectional, moving from the *melpomene* to the silvaniform clade and vice versa. The history of gene exchange has also been complex, with contributions from multiple silvaniform species in addition to *H. besckei*. We also detect a signature of ancient introgression of the entire Z chromosome between the silvaniform and *melpomene*/*cydno* clades.

**Conclusions:**

Our study provides a genome-wide portrait of introgression between distantly related butterfly species. We further propose a comprehensive and efficient workflow for gene flow identification in genomic data sets.

**Electronic supplementary material:**

The online version of this article (doi:10.1186/s13059-016-0889-0) contains supplementary material, which is available to authorized users.

## Background

Hybridization, or interbreeding between species, has the potential to influence adaptation and speciation in a variety of ways. For instance, occasional hybridization between incompletely isolated species may contribute to either accelerating speciation or breaking down species barriers [1]. In addition, hybridization can also lead to adaptive introgression by transmitting beneficial alleles between species via backcross hybrids [2–4], which can happen during either sympatric speciation or the secondary contact phase of allopatric speciation. There are a number of striking examples of adaptive introgression in plants, both between hybridizing wild species as well as between crops and their wild relatives [5–7]. Furthermore, there is a growing list of examples of adaptive introgression in animals. For instance, an allele associated with rodenticide warfarin resistance at the gene *Vkorc1* has been shared by mouse species [8], and an insecticide resistance mutation has been transferred between *Anopheles* sibling species [9, 10]. Similarly, haplotypes at the *ALX1* gene, which is strongly associated with beak shape in Darwin’s finches, appear to be shared among species due to hybridization [11]. In addition, recent examples suggest modern humans have benefited by adaptive introgression from different populations and extinct species: introgressed Neanderthal alleles may have helped modern humans adapt to non-African environments [12, 13], and high-altitude adaptive traits have been contributed to Tibetans from Denisovans and Nepalese Sherpa [14, 15].

**Fig. 1:**
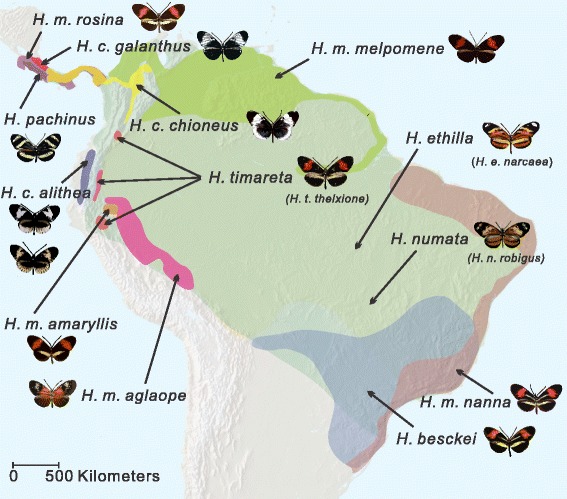
The geographical distribution of *Heliconius* butterfly species. The distributions of *Heliconius melpomene-cydno-timareta* and silvaniform clades are shown in different colors, along with wing pattern images of focal taxa

Another striking example of adaptive introgression in animals involves wing pattern mimicry in *Heliconius* butterflies [16, 17]. The genus *Heliconius* is a group of neotropical butterflies that display diverse Müllerian mimetic wing patterns to warn predators of their toxicity [18, 19]. Given a long history of research and rapidly developing genomic resources, *Heliconius* is also an excellent system to address a variety of evolutionary questions related to adaptation and hybridization [20–25]. The evolution of mimicry in *Heliconius* has resulted from introgression between closely related species as well as convergent evolution between more distantly related species. For instance, *Heliconius* species belonging to the *melpomene-cydno-timareta* clade, which hybridize regularly but are subject to Haldane’s rule, share wing color patterns due to introgression between species [16, 17, 26–28]. On the other hand, between distantly related co-mimetic pairs, such as between *Heliconius melpomene* and *Heliconius erato*, nearly identical wing patterns have evolved independently [29–31]. Between these two extremes are rare instances of mimicry between the *melpomene-cydno-timareta* and silvaniform clades; these clades are ecologically and morphologically distinct and hybridization between them is rare [32, 33]. Previously, gene flow has been documented from the *melpomene-cydno-timareta* clade into the silvaniform clade [34], and *Heliconius elevatus*, a silvaniform species that displays a *melpomene*-like rayed color pattern was shown to have acquired its mimicry phenotype via introgression from *H. melpomene* [16]. One of the most widespread color patterns across the broad distribution of co-mimics *H. melpomene* and *H. erato* is the red and yellow banded ‘postman’ phenotype seen throughout Central America, much of Brazil and isolated patches in Peru and Colombia. In coastal Brazil, *H. melpomene nanna* and *H. erato phyllis* co-occur with the only silvaniform species that shares this postman color pattern, *Heliconius besckei* (Fig. 1). The origin of mimicry in *H. besckei* remains elusive, however, and given rare ongoing hybridization among *melpomene* and silvaniform group species, could have been the product of introgression or convergent evolution.

Recently, next generation sequencing technologies have enabled genome-wide studies to depict global introgression patterns in a variety of systems [11, 16, 35–38]. As a result, a diverse set of statistical tests have been developed that utilize genome-wide data for the detection of introgression between closely related species. For instance, the four taxon ABBA-BABA test was originally designed to detect interbreeding between modern humans and Neanderthals [35, 39] and then was widely applied to test for ancient admixture among other systems such as *Heliconius* and swallowtail butterflies, spiny lizards, *Zimmerius* flycatchers and crows [16, 40–43]. Here, we examine genome-wide patterns of divergence and introgression in *Heliconius* butterflies using full genome resequencing data, focusing on potential adaptive introgression between *H. melpomene* and *H. besckei*. We integrate a series of population genetic and phylogenetic approaches to infer introgression and propose a comprehensive and efficient workflow for gene flow identification in large population genomic data sets. Our results provide a genome-wide portrait of polarized introgression between distantly related species, yielding multiple discrete instances of putative adaptive introgression.

## Results

### Whole genome phylogenetic analyses

**Fig. 2:**
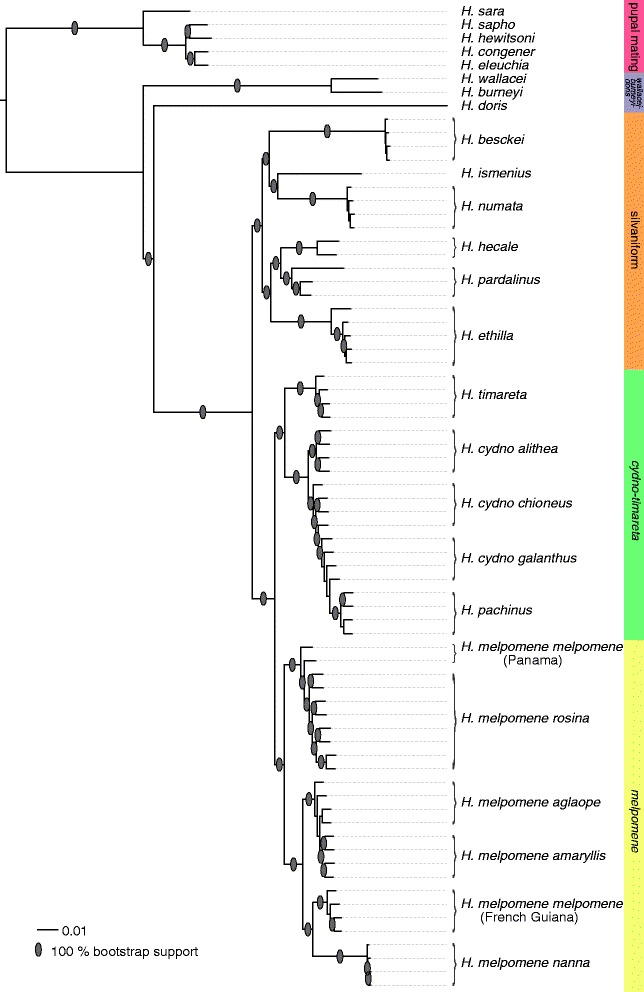
Genome-wide phylogeny of *Heliconius* butterflies. The maximum-likelihood phylogenetic tree is based on the genome-wide SNP data (23 Mb alignment) with the clades indicated as color-coded bars. The scale bar represents the percentage of substitutions per site

We first characterized the evolutionary history of *Heliconius* butterflies using genome-wide single nucleotide polymorphism (SNP) data (approximately 23 million SNPs) from 73 butterflies representing 29 species (Fig. 2). This analysis was used to define a sister taxon for *H. besckei* for introgression scans between *H. besckei* and *H. melpomene nanna* using the *D*-statistic. In Brazil, *H. besckei* co-occurs with silvaniform species *Heliconius numata robigus* and *Heliconius ethilla narcaea* but our results strongly supported *H. numata* + *Heliconius ismenius* as the sister clade to *H. besckei* so we selected *H. besckei*, *H. numata* and *H. m. nanna* as the three ingroup taxa for Patterson’s *D*-statistic tests. Moreover, the time-calibrated maximum-likelihood tree of 30 samples showed that the split between *H. besckei* and *H. numata* was estimated at 1.93 million years ago (Mya), which was earlier than the split between the *Heliconius cydno* and *H. melpomene* lineages (1.34 Mya) (Figure S1 in Additional file 1). This result suggests that if introgression occurred between *H. besckei* and *H. m. nanna*, it could have happened at one or more distinct time points: between *H. besckei* and the *melpomene*-*cydno-timareta* ancestor, between *H. besckei* and the *melpomene* ancestor, and/or between *H. besckei* and *H. m. nanna*.

### Searching for large-scale introgression between *H. m. nanna* and *H. besckei*

Since *H. m. nanna* and *H. besckei* are sympatric species that are potentially interfertile and display similar wing patterns, our a priori hypothesis was that wing pattern mimicry was shared via introgression, either originating in *H. melpomene* and then being passed to the ancestor of *H. besckei* or vice versa. To test this hypothesis, we calculated Patterson’s *D*-statistic incorporating *H. numata* as the third ingroup taxon and *Heliconius wallacei* as the outgroup taxon. We used *H. wallacei* as the outgroup because this sample had the most genotype calls among the three potential outgroup samples, *H. wallacei*, *Heliconius burneyi* and *Heliconius doris* (Table S1 in Additional file 2). For each fixed 50 kb window across 21 chromosomes, we estimated derived SNP allele frequencies supporting either ‘ABBA’ or ‘BABA’ patterns among the ingroup taxa and then calculated the mean *D*-statistic value for each chromosome (Table S2 in Additional file 2). The results suggested no large-scale gene flow between *H. besckei* and *H. m. nanna*, but rather, greater gene flow between *H. numata* and *H. m. nanna*, although the *D*-statistic values were close to zero (Table S2 in Additional file 2). This marginal but significant *D*-statistic between *H. numata* and *H. m. nanna* was unexpected. Given the large overlap in the distributions of *H. numata* and *H. melpomene*, it is possible that these two species do exchange more gene flow than between *H. besckei* and *H. melpomene*.

### Evidence of gene flow at the *B*/*D* mimicry locus

**Fig. 3:**
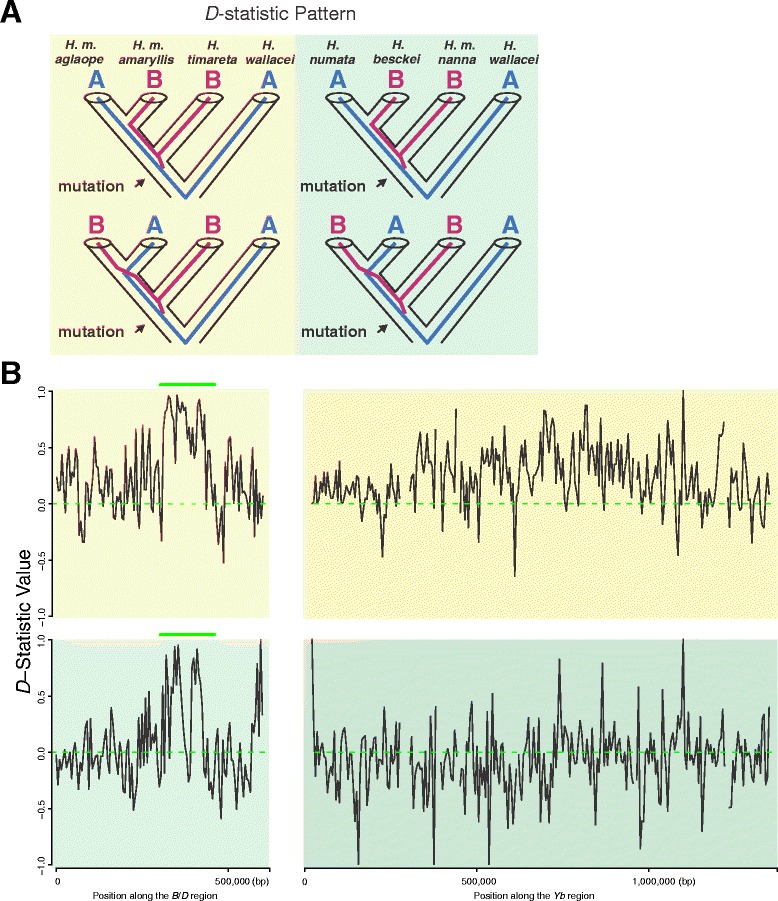
Patterson’s *D*-statistic scans along the *B*/*D* and *Yb* mimicry loci scaffolds. For the tree topology above (**a**), clear introgression patterns (labeled with *green bars*) are observed along *B*/*D* between *H. m. amaryllis* and *H. timareta* and between *H. besckei* and *H. m. nanna*, using 5 kb adjacent windows (**b**)

*Heliconius* wing patterning is controlled by a small number of Mendelian switch loci with the red forewing band and yellow hind wing band of the *H. melpomene* postman phenotype specifically controlled by the *B*/*D* and *Yb* loci, respectively [44, 45]. Previous analysis showed evidence of introgression around the *B*/*D* and *Yb* mimicry loci between co-mimetic postman as well as rayed *H. melpomene* and *Heliconius timareta* [16]. We applied a 5 kb window size to plot *D*-statistic values along *B*/*D* and *Yb* genomic intervals to infer potential gene flow between *H. besckei* and *H. m. nanna*. For comparison, we also plotted *D*-statistic values along *B*/*D* and *Yb* genomic intervals in co-mimetic *Heliconius melpomene amaryllis* and *H. timareta* (see also [16]). The results revealed two sharp peaks of elevated *D* in the *B*/*D* interval indicating genetic similarity between *H. besckei* and *H. m. nanna*, and the physical position of this region coincided perfectly with the genomic region showing evidence of introgression between *H. m. amaryllis* and *H. timareta* (Fig. 3). Statistical tests using a fixed 5 kb window through the *B*/*D* interval were significantly different from zero (*D*_*numata*, *besckei*, *m. nanna*, *wallacei*_ = 0.385 ± 0.109, *P* = 0.0004), indicative of a strong introgression signal at this mimicry locus between *H. besckei* and *H. m. nanna*. The *Yb* locus, in contrast, showed no clear evidence of elevated *D*, and this region also showed a more diffuse signature of allele sharing in the *H. m. amaryllis* and *H. timareta* comparison (Fig. 3). Statistical tests through the region of the *Yb* locus that showed the strongest evidence of introgression in the *H. m. amaryllis* and *H. timareta* comparison (500 kb to 1 Mb) revealed a pattern similar to the genomic background, with greater allele sharing between *H. numata* and *H. m. nanna* in comparison with *H. besckei* and *H. m. nanna* (*D*_*numata*, *besckei*, *m. nanna*, *wallacei*_ = −0.084 ± 0.034, *P* = 0.01318). These results suggest wing pattern mimicry, but perhaps only variation at the *B*/*D* mimicry locus, was introgressed between *H. melpomene* and *H. besckei*.

### Patterns of genome-wide introgression

To further examine genome-wide patterns of introgression between *H. m. nanna* and *H. besckei*, we calculated the *D*-statistic for every 5 kb, 10 kb and 50 kb window across the genome separately and used the maximum signature of putative *H. besckei–H. m. nanna* introgression at *B*/*D* as a minimum threshold (*D*_*numata*, *besckei*, *m. nanna*, *wallacei*_ ≥ 0.74 for 5 kb window, *D*_*numata*, *besckei*, *m. nanna*, *wallacei*_ ≥ 0.56 for 10 kb window, *D*_*numata*, *besckei*, *nanna*, *wallacei*_ ≥ 0.34 for 50 kb window). We kept candidate loci only if supported by more than five valid SNPs per 5 kb, which yielded 97 unique candidate introgression loci: 60 5-kb segments, 32 10-kb segments and 5 50-kb segments (Table S3 in Additional file 2). Next, we calculated the *f-*statistic for the 97 focal intervals and extracted those with *D* and *f*_*d*_ significantly different from zero (*P* < 0.01; Table S3 in Additional file 2). The *f-*statistic was originally designed to estimate the proportion of the genome exchanged as a result of introgression [35], but it was recently modified to complement the *D*-statistic in identifying introgressed genomic segments [46]. A total of 50 candidate introgression loci were significant with both the *D* and *f*_*d*_ tests (Table S3 in Additional file 2).

In order to help distinguish between introgression and ancestral variation, we calculated DNA sequence divergence (d_xy_) for each candidate introgression interval and compared this with chromosomal mean d_xy_ (Table S4 in Additional file 2) because introgressed regions generally show lower absolute genetic divergence [47]. A total of 91 loci showed lower *H. besckei*–*H. m. nanna* d_xy_ compared with neighboring regions and their chromosome as a whole (Table S5 in Additional file 2). Martin et al. [46] showed that d_xy_ can be correlated with *D* due to factors other than gene flow so we further tested for evidence of allele sharing between *H. besckei* and *H. m. nanna* by comparing *H. besckei*–*H. m. nanna* d_xy_ to that between other species comparisons, *H. besckei*–*H. numata*, and *H. m. nanna*–*H. numata.* We found that at 39 of 97 intervals, *H. besckei*–*H. m. nanna* d_xy_ was lower than both the silvaniform comparison, *H. besckei*–*H. numata*, and the *H. m. nanna*–*H. numata* comparison (Table S5 in Additional file 2), again pointing to allele sharing between *H. besckei* and *H. melpomene*.

We also checked sequencing read depths for all candidate introgression loci among the four *D*-statistic taxa to rule out false positives caused by potential read mapping artifacts (Table S6 in Additional file 2). For instance, a signature of allele sharing between taxa could result from a subset of reads most similar to the reference genome, *H. melpomene* in this case, mapping back to the reference better than more divergent reads. This read mapping artifact could produce an apparent signature of introgression between species (specifically *H. melpomene* introgression into other species) but it should also yield reduced read coverage in the affected area. On the other hand, unusually high read depth might be due to read misalignment and could influence allele frequency estimates. Based on the sample statistics shown in Tables S1 and S6 in Additional file 2, loci with high (above 40) or low read depth (below 5) were filtered out. In total, we found that 85 candidate introgression loci were within an acceptable sequencing coverage range. By overlaying the results from all statistics, we identified a subset of 41 candidate introgression loci, including two adjacent 50 kb regions from the *B*/*D* mimicry locus, that were supported by all measures (Table 1). These genomic regions were distributed among 16 of the 21 chromosomes in *H. melpomene*. Many of the 5 kb segments were filtered out but most of the 10 kb and 50 kb segments were retained, which indicates a relatively robust introgression pattern of these larger genomic regions.

**Table 1:** *D*-statistics and *f*_*d*_-statistics for 41 well-supported candidate introgression loci

ID	Chromosome	Scaffold	Length (kb)	*D*	ID	Chromosome	Scaffold	Length (kb)	*D*
1	chr1:6425000-6430000	HE669357:205937-210937	5	0.7961	73	chr12:850000-860000	HE671156:128477-138477	10	0.8058
4	chr3:2395000-2400000	HE671395:17962-22962	5	0.7956	74	chr12:5030000-5040000	HE671858:12754-22754	10	0.6685
6	chr3:6965000-6970000	HE670746:58196-63196	5	0.8694	75	chr12:15840000-15850000	HE670557:425233-435233	10	0.6485
7	chr4:700000-705000	HE672065:10133-15133	5	0.7772	76	chr13:280000-290000	HE670462:42188-52188	10	0.5986
8	chr4:1895000-1900000	HE669256:275358-280358	5	0.8587	77	chr14:890000-900000	HE671459:112697-122697	10	0.6072
11	chr5:6580000-6585000	HE669934:5702-10702	5	0.8255	79	chr16:320000-330000	HE671439:139401-149401	10	0.5721
17	chr6:11110000-11115000	HE671186:279186-284186	5	0.8347	80	chr16:9020000-9030000	HE671226:97586-107586	10	0.6141
18	chr6:12485000-12490000	HE671449:40866-45866	5	0.8090	82	chr18:140000-150000	HE671865:140000-150000	10	0.5920
27	chr10:300000-305000	HE671375:300000-305000	5	1.0000	84	chr18:14400000-14410000	HE671975:123370-133370	10	0.5955
34	chr12:6280000-6285000	HE671289:156614-161614	5	0.8641	85	chr18:14410000-14420000	HE671975:133370-143370	10	0.6745
37	chr13:4785000-4790000	HE671822:93367-98367	5	0.7680	87	chr19:9790000-9800000	HE669843:33987-43987	10	0.6067
41	chr13:11675000-11680000	HE671818:16514-21514	5	0.7586	88	chr19:11320000-11330000	HE670722:35821-45821	10	0.6500
53	chr18:635000-640000	HE671969:330491-335491	5	0.7548	89	chr19:11770000-11780000	HE670829:29944-39944	10	0.5737
54	chr18:5425000-5430000	HE670289:23055-28055	5	0.8661	91	chr20:3980000-3990000	HE670907:120779-130779	10	0.5861
57	chr19:6870000-6875000	HE672046:136-5136	5	0.7523	92	chrZ:210000-220000	HE672038:210000-220000	10	0.5970
61	chr1:8850000-8860000	HE672073:240785-250785	10	0.6122	93	chr1:12150000-12200000	HE667922:62931-112931	50	0.3897
62	chr1:15010000-15020000	HE671914:85876-95876	10	0.5800	94	chr11:9800000-9850000	HE672060:343196-393196	50	0.4483
63	chr2:150000-160000	HE671404:40206-50206	10	0.5662	95	chr11:9850000-9900000	HE672060:393196-443196	50	0.7113
65	chr2:1880000-1890000	HE672041:90176-100176	10	0.6762	96	chr18:1150000-1200000	HE670865:305844-355844	50	0.4883
71	chr10:11830000-11840000	HE671341:728179-738179	10	0.5729	97	chr18:1200000-1250000	HE670865:355844-405844	50	0.3468
72	chr12:840000-850000	HE671156:118477-128477	10	0.6338					

### Time and direction of gene flow among *Heliconius* species

**Fig. 4:**
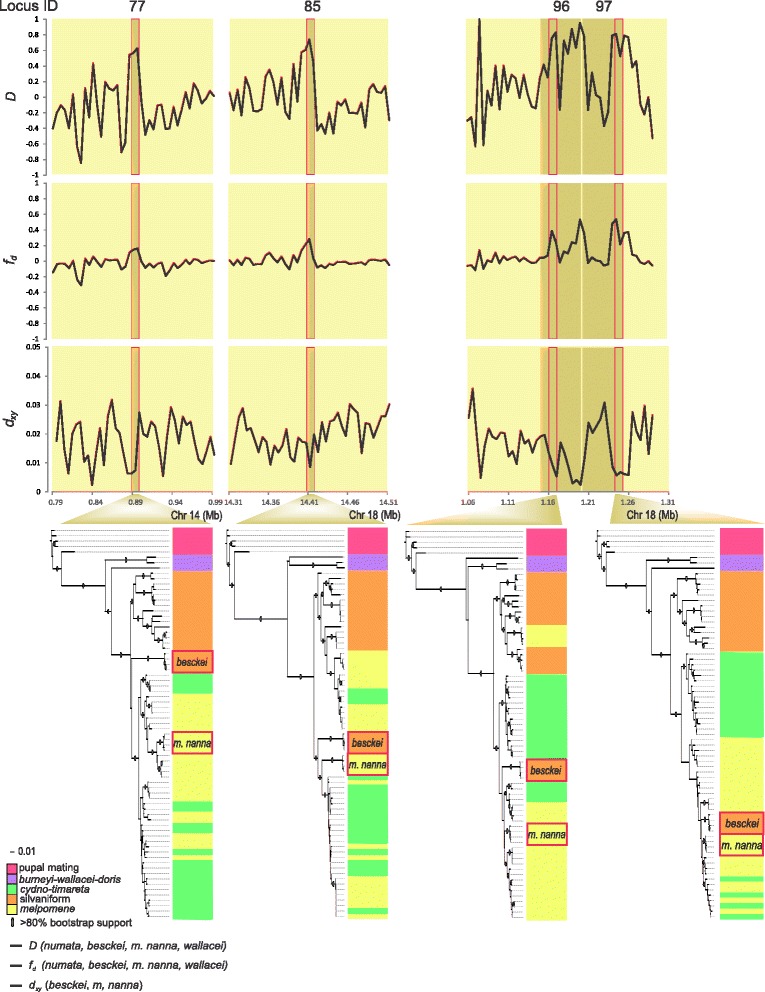
Signatures of introgression between *H. besckei* and *H. m. nanna* along four candidate introgression loci. For each candidate locus, the Patterson’s *D*-statistic, *f*_d_ and d_xy_ scans are performed and the maximum likelihood phylogenetic tree using 10 kb window size is constructed to infer the timing and direction of gene flow

According to our phylogenetic analysis, *H. besckei* originated prior to diversification within the *melpomene-cydno-timareta* clade so introgression could have occurred before or after the origin of *H. m. nanna.* Furthermore, because evidence of introgression emerged using relatively small (5 kb, 10 kb and 50 kb) window sizes, it is likely that gene flow between *H. besckei* and *H. melpomene* was relatively ancient, so that signals of longer introgressed haplotypes were lost over time due to recombination and subsequent substitutions. To trace the history of introgression and infer the direction of gene flow, we reconstructed genealogies for each 5 kb and 10 kb candidate introgression locus using both the original window and a larger window size (original + 5 kb on either side for 5 kb candidate loci, original + 10 kb on either side for 10 kb candidate loci). This approach was motivated by the need to both maximize phylogenetic signal, potentially lacking in small genomic segments, and determine the physical extent of introgressed haplotypes. For each 50 kb candidate introgression loci, we reconstructed genealogies for the original 50 kb as well as 10 kb subsections (Fig. 4; Figures S2–S9 in Additional file 1).

We used the relationships of taxa on gene genealogies of candidate introgression loci, and discordance from the genome-wide tree, to infer the history of gene flow at each locus. For instance, visual inspection of the genealogy for candidate introgression locus 77 places all *H. besckei* haplotypes as a sister-clade to all *melpomene-cydno-timareta* haplotypes (Fig. 4). From this relationship we inferred that there was ancient gene flow between *H. besckei* and the common ancestor of the *melpomene-cydno-timareta* clade (Table 2), but because this is the oldest time point and we lack another clade between these taxa and the outgroup, we could not infer the direction of gene flow. In contrast, for candidate introgression loci 96 and 97, which are from the *B*/*D* mimicry locus, *H. besckei* haplotypes clustered not with other silvaniform species, but with a subset of *melpomene-cydno-timareta* clade species (Fig. 4). From this we inferred that introgression occurred from *H. melpomene* into *H. besckei* and that it occurred historically (Table 2), prior to some diversification among *H. melpomene* subspecies, since this haplotype is shared among a number of *H. melpomene* subspecies (Fig. 4). Inferring the history of introgression in this way requires that the inferred genealogy is discordant from the genome-wide tree, with respect to the focal taxa, but that discordance across the tree as a whole is not so pervasive as to make the historical pattern uninterpretable. Therefore, we restricted these analyses to candidate introgression loci for which gene genealogies showed discordance specific to the focal taxa but otherwise limited discordance over the rest of the tree. Gene trees for 25 of 44 candidate introgression loci met these criteria (Fig. 4; Figures S2–S9 in Additional file 1) and we were able to infer the timing and direction of gene flow for 23 of them (Table 2).

**Table 2:** Inferring the timing and direction of gene flow at 23 candidate introgression loci

ID	Location	Direction of gene flow
61^a,b^	chr1:8850000-8860000	*H. ethilla* to *H. m. nanna*
93	chr1:12150000-12200000	*H. melpomene ancestor* to *H. besckei*
63^a^	chr2:150000-160000	*H. ethilla* to *H. m. nanna*
65^a^	chr2:1880000-1890000	*H. ethilla* to *H. m. nanna*
7^b^	chr4:700000-705000	*H. pardalinu*s to *H. m. nanna* and *H. m. melpomene* (FG) ancestor
17^b^	chr6:11110000-11115000	*H. besckei* or *H. ismenius* to *H. m. nanna*
27^a,b^	chr10:300000-305000	*H. pardalinu*s to *H. melpomene* ancestor
71^a,b^	chr10:11830000-11840000	*H. hecale* to *H. m. nanna*
94^a,b^	chr11:9800000-9850000	*H. ethilla* to *H. m. nanna*
95	chr11:9850000-9900000	One of the silvaniform species to *H. m. nanna*
72^a^	chr12:840000-850000	*H. ethilla* to *H. m. nanna*
73^a,b^	chr12:850000-860000	*H. ethilla* to *H. m. nanna*
75^a,b^	chr12:15840000-15850000	*H. ethilla* to *H. m. nanna*
76^a,b^	chr13:280000-290000	*H. pardalinu*s to *H. melpomene* ancestor
77^b^	chr14:890000-900000	Between *H. besckei* and *melpomene-cydno-timareta* ancestor
79^a^	chr16:320000-330000	Between *H. besckei* and *melpomene-cydno-timareta* ancestor
80^a,b^	chr16:9020000-9030000	*H. hecale* to *H. melpomene* ancestor
82^a,b^	chr18:140000-150000	*H. besckei* to *H. m. nanna*
96^b^	chr18:1150000-1200000	*H. melpomene* ancestor to *H. besckei*
97^b^	chr18:1200000-1250000	*H. melpomene* ancestor to *H. besckei*
84^b^	chr18:14400000-14410000	*H. m. nanna* to *H. besckei*
85^a,b^	chr18:14410000-14420000	Between *H. besckei* and *melpomene-cydno-timareta* ancestor
91^b^	chr20:3980000-3990000	*H. pardalinus* to *H. melpomene* ancestor

^a^ Inference is supported by phylogenetic bootstrap values > 80

^b^ Inference is supported by additional *D*-statistics with *P* values < 0.05

We were surprised to find that inspection of many trees suggested potential gene flow between *H. melpomene* and silvaniform species other than *H. besckei*. For instance, trees for loci 61, 73, 75, and 94 clustered *H. m. nanna* with *Heliconius ethilla* as opposed to *H. besckei* (Figures S3, S4 and S7 in Additional file 1). All of our genome-wide analyses used *H. besckei*’s closest relative, *Heliconius numata*, for comparison but other silvaniform species also co-occur in this Brazilian community and could be exchanging genes with the focal taxa as well. It is possible that some of the strong signatures of putative *H. besckei–H. m. nanna* introgression could actually be a result of introgression from another silvaniform species. Therefore, we calculated *D*-statistics to test potential gene flow between *H. m. nanna* and silvaniform species such as *H. ethilla*, *Heliconius pardalinus*, *Heliconius hecale* and *H. ismenius* for 23 candidate introgression loci (Table S7 in Additional file 2). These results suggest that other silvaniform species have indeed been involved in as many as two-thirds of the inferred introgression events (Table 2). Furthermore, the dynamics appear to differ between introgression events involving *H. besckei* versus those involving other silvaniform species (Table 2). Specifically, all introgression involving *H. besckei* appears to have been in the direction of gene flow into *H. besckei*. These events also appear to be older, often involving the *H. melpomene* ancestor or the *melpomene-cydno-timareta* ancestor. In contrast, introgression involving all other silvaniform species appears to move in the opposite direction, into *H. melpomene*, and is often inferred to have occurred at more recent time points, frequently involving *H. m. nanna* specifically (Table 2).

### Annotation of introgression loci

We found that protein coding genes were located in 32 of 41 putatively introgressed loci (Table S8 in Additional file 2), supporting the idea that introgression may have functional consequences. The list of annotated genes contained in the candidate introgression loci suggests a number of interesting biological functions potentially associated with introgression, including collagen and cuticle matrix formation, metabolism, embryonic patterning, synapse function, and heat stress, to name a few. Furthermore, the candidate introgression loci that do not contain protein coding sequences are likely to have functional consequences as well. A clear example of this is the strong signature of introgression we detected between *H. melpomene* and *H. besckei* at the *B*/*D* mimicry locus. This region on chromosome 18 does not contain genes but it is immediately adjacent to the gene *optix*, which is known to control all red color pattern variation across *Heliconius* butterflies [44]. This is the same genomic region that has been introgressed among co-mimetic *H. melpomene*, *H. timareta*, and *H. elevatus* in Peru and Colombia and contains the regulatory elements governing differential expression of *optix* [16, 17].

### Analysis of the Z chromosome

Previous work comparing *H. cydno* and *H. melpomene* found pronounced genetic differentiation and greatly reduced interspecific gene flow on the Z chromosome, relative to autosomes [48, 49], a population genetic pattern consistent with the large Z effect on hybrid female sterility in crosses between these two species [50], as well as in other *Heliconius* butterflies [51], and the disproportionately large role of the Z chromosome in mediating reproductive isolation in female heterogametic taxa in general [52]. We compared *H. m. nanna* with *H. besckei* and *H. numata* and also found reduced introgression on the Z chromosome relative to autosomes (Table S2 in Additional file 2). However, comparisons between *H. m. nanna* and other silvaniform species, such as *H. ethilla*, revealed a distinct and unexpected signature of introgression on the Z chromosome, apparent as highly elevated *D* (Table S9 in Additional file 2). In addition to elevated *D* on the Z chromosome, this signature also appeared as reduced d_xy_ and F_ST_ on the Z chromosome relative to comparisons between other species (Table S9 in Additional file 2), although the F_ST_ results were not as striking as the other two statistics. Also, Z chromosome F_ST_ values were still generally higher than autosomes, consistent with the idea that this Z chromosomal introgression was relatively old. By swapping taxa in the four-taxon *D*-statistic test, we found that the signature of shared variation on the Z chromosome was not exclusive to *H. m. nanna* and *H. ethilla* but could be traced back to sharing between the entire *melpomene-cydno-timareta* clade and the silvaniform subclade that includes *H. ethilla*, *H. hecale*, and *H. pardalinus* (Table S9 in Additional file 2). Furthermore, a genealogy based on all Z chromosome SNP variation revealed a deep discordance with the genome-wide tree with the *H. ethilla*, *H. hecale*, and *H. pardalinus* clade sister to the *melpomene-cydno-timareta* clade, as opposed to the other silvaniform clade (Figure S10 in Additional file 1). As a whole, these results suggest ancient introgression of the entire Z chromosome between just one of these two silvaniform subclades and the ancestor of the entire *melpomene-cydno-timareta* clade.

## Discussion

### The fate of introgressed genetic variation

Gene flow between species can play diverse and even opposing roles in the evolutionary process, from homogenizing genetic variation and eroding the species barrier to providing novel alleles that facilitate adaptation. Phylogenetic discordance we see today is the result of interactions among multiple processes including sorting of ancestral variation, introgression, and recombination over the combined history of two related species. Hybridization and introgression offer the opportunity for exchange of genetic material and ultimately selection plays a central role in the integration of introduced alleles into the recipient genome. For instance, introgression of a deleterious allele is liable to be prevented, while a neutral allele should undergo random recombination and accumulate further neutral substitutions over time and has a low probability of fixation, and a beneficial allele should spread in the new population with a higher probability of fixation. The length of introgressed haplotypes also provides information about the timing of gene flow and the pace of selective sweeps because recombination and subsequent substitutions will erode them over time. Overall, therefore, we expect that small, introgressed genomic regions originating from the oldest gene flow events are likely to be adaptive.

In this study, we found various instances consistent with historical adaptive introgression between *H. melpomene* and *H. besckei* in Brazil. Our analyses suggest little recent gene flow between these taxa and point to gene exchange at time points older than when *H. melpomene aglaope* donated alleles at the *B*/*D* mimicry locus to *H. timareta florencia*, which could be detected at a chromosome-wide level [16]. We also found that protein coding genes are located in a majority of putatively introgressed loci (Table S8 in Additional file 2). Furthermore, a number of the inferred introgression events occurred between other silvaniform species and *H. melpomene*, which suggests a more widespread pattern of gene flow among these lineages, not only limited to co-mimics that share a similar wing pattern.

### Potential functional consequences of introgression

The persistence of small, apparently introgressed genomic regions over time is suggestive of a functional role, although it is often difficult to determine the exact nature of those roles given our limited knowledge of butterfly functional genetics. In the case of mimicry, our data suggest that *H. besckei* acquired its mimetic wing pattern from *H. melpomene*. Our analyses reveal one other genomic region that appears to share the same history as the *B*/*D* mimicry locus on chromosome 18, this being a 50 kb interval on chromosome 1 (locus 93) that we infer to have been transferred along with wing pattern mimicry from the *H. melpomene* ancestor to *H. besckei* (Table 2). This candidate introgression locus contains collagen type IV subunit α-2 and collagen type IV subunit α-1 lies right outside this candidate locus. Collagen IV is the main constituent of basement membranes and plays an active morphogenetic role in determining organ shape and animal form [53]. Furthermore, collagen type IV subunits α-1 and α-2 play important roles in muscle function across animals and they have recently been implicated in flight muscle function in the migratory monarch butterfly, *Danaus plexippus* [54]. Mimicry in *Heliconius* butterflies is well known to involve flight behavior in addition to wing pattern. Srygley and colleagues have shown that aspects of flight kinematics, primarily wing-beat frequency [55, 56], converge among co-mimetic *Heliconius* species, as do wing and body morphology associated with biomechanics [57, 58]. While highly speculative, our genomic data offer the intriguing possibility that *H. besckei* may have acquired a behavioral component of mimicry, locomotor mimicry, via introgression from *H. melpomene* in addition to wing pattern mimicry. This hypothesis will require further functional tests in the future.

### A unique evolutionary signature on the Z chromosome

Sex-linked genes often play an important role in generating reproductive isolation between closely related species [52, 59] and therefore sex chromosomes might generally be expected to experience reduced interspecific gene flow in comparison with autosomes. Consistent with this, previous studies on nightingales and *Heliconius* species in the *cydno-melopmene* clade found a pattern of reduced introgression on the Z chromosome [49, 60]. Similarly, reduced gene flow on the X chromosome was recently documented in *Anopheles* mosquitoes where autosomal introgression is pervasive [38]. Our analysis of Z chromosome variation among more distantly related *Heliconius* species revealed a surprising result. Comparison of *H. melpomene* and *H. besckei* showed the expected pattern of reduced introgression on the Z chromosome relative to autosomes. However, comparisons with other silvaniforms suggest ancient introgression of the entire Z chromosome between the silvaniform subclade that contains *H. hecale*, *H. pardalinus*, and *H. ethilla*, and the ancestor of the *melpomene-cydno-timareta* clade.

### Efficiency of detecting introgressed loci

The methods for generating high-density SNP data in virtually any organism are now relatively straightforward, and with this new technology come statistical tools to examine genome-wide patterns of introgression. Established analytical pipelines for examining gene flow between species include both divergence-based genome scans (linkage disequilibrium, F_ST_ and *D*-statistic) and likelihood/model-based methods [61, 62]. Given that our sampling fit the requirements of the *D*-statistic (two sister species, a third species potentially involved in introgression, and an outgroup species), our goal was to integrate *D*-statistic, *f*-statistic, and DNA sequence divergence approaches to infer introgression and disentangle incomplete lineage sorting from gene flow. In order to maximize the power of our approach, we further defined a baseline of expected signatures of introgression using the observed patterns around the *B*/*D* mimicry locus. This is a genomic region for which we had an a priori hypothesis and for which the data yielded a strong signature of introgression. Finally, we used phylogenetics, comparing trees inferred using genome-wide SNP data with those from candidate introgression loci, to infer the timing and direction of introgression between species. Here we applied a progressive phylogenetic analysis with multiple window sizes which enabled us to characterize the introgression histories for 23 of 41 loci. Importantly, our pipeline offers an efficient solution to identify and polarize genome-wide introgression events that can be applied widely to emerging genome-wide polymorphism data.

## Conclusions

Previous work has shown that closely related *Heliconius* species that mimic one another evolved that similarity by exchanging wing patterning alleles through hybridization. However, the potential for adaptive introgression across the rest of the genome has not been explored, nor has the extent of gene flow among more distantly related species. Here we focused on *H. besckei*, an enigmatic species from Brazil that has a color pattern that matches sympatric co-mimics but differs completely from its closest relatives, making it a good candidate for introgression of wing pattern mimicry. We tested this and found a strong signature of introgression at the gene *optix*, which controls red wing patterning in *Heliconius*, and then we used these population genetic signatures as a minimum threshold for genome-wide comparisons. This approach yielded 39 additional genomic regions that also showed strong evidence of introgression. Analysis of these putatively introgressed genomic regions revealed that gene flow has been on-going, bi-directional between clades, and complex, involving multiple extant species as well as their ancestors. We also found evidence of ancient introgression of the entire Z chromosome between lineages, which is unexpected because this chromosome has previously been shown to be resistant to introgression between closely related *Heliconius* species. As a whole, our results substantially expand the potential impact of introgressive hybridization throughout the evolutionary history of *Heliconius* butterflies.

## Methods

### Sample preparation and sequencing

Twenty-five adult butterflies were collected in the field in Brazil, Ecuador and Costa Rica. For each individual, wings were carefully separated and genomic DNA was extracted from thoracic tissue using a DNeasy Blood & Tissue Kit (Qiagen). Illumina paired-end libraries were constructed using the Illumina Truseq protocol and then were pooled and sequenced using an Illumina HiSeq2000. Raw reads were demultiplexed according to their barcodes.

### Data collection and genotyping calling

We downloaded 48 available whole genome resequencing datasets from NCBI (PRJNA226620) [48, 63] and ENA (ERP002440) [49]. Low quality reads with fewer than 90 % bases that had a minimum quality score above 10 were removed after quality filtering from 73 genome resequencing datasets and the rest were aligned to the *H. melpomene* v1.1 [16] using Bowtie2 v2.0.0-beta7 [64] with parameter -very-sensitive-local and then re-ordered and sorted by Picard v1.84 (http://broadinstitute.github.io/picard/). PCR duplicates were removed using Picard. RealignerTargetCreator and IndelRealigner [65] in GATK v2.1 were used to realign indels and UnifiedGenotyper [66] was used to call genotypes across 73 individuals using the following parameters: heterozygosity 0.01, stand_call_conf 50, stand_emit_conf 10, dcov 250. SNPs with good quality (Qual > 30) were finally used in the subsequent analyses (Table S1 in Additional file 2).

### Genome-wide phylogeny and divergence time estimation

Polymorphism genotype calls existing in all 73 individuals with good quality (around 23.14 Mb) were aligned and converted into PHYLIP format and a genome-wide maximum-likelihood phylogenetic tree was constructed using RAxML [67] with the GTRGAMMA model and 100 bootstrap replicates. The tree image was created using iTOL [68]. A genome-wide tree topology including 32 taxa was extracted as input for the software PhyTime [69], which was used to estimate divergence times, calibrated using the mean split time estimates between *H. cydno* and *H. melpomene* (1.4 Mya) and between *H. cydno* and *H. pachinus* (0.43 Mya) from a previous population genomics study [48]. The PhyTime output tree was processed using TreeAnnotator [70]. For candidate introgression loci, maximum-likelihood trees were generated using PhyML3.0 [71] with the GTR model and 100 bootstrap replicates to infer the direction of gene flow.

### Detecting gene flow among *H. besckei*, *H. numata* and *H. m. nanna*

We integrated both Patterson’s *D*-statistic [35, 39] and a modified *f*-statistic (*f*_d_) [46] to better identify potential introgressed loci across the whole genome among three ingroup taxa (*H. m. nanna*, *H. besckei* and *H. numata*) using *H. wallacei* as an outgroup. The *D*-statistic was used to examine the phylogenetic distribution of derived alleles at loci that display either an ABBA or BABA allelic configuration (Fig. 2). Since we had four individuals per taxon, the frequency of the derived allele at each site in each population was used instead of binary counts of fixed ABBA and BABA sites with [39]:

1 $$ D\left({P}_1,{P}_2,{P}_3,O\right)=\frac{{\displaystyle \sum_{i=1}^n\left[\left(1-\widehat{P_{i1}}\right)\widehat{P_{i2}}\widehat{P_{i3}}\left(1-\widehat{P_{i4}}\right)-\widehat{P_{i1}}\left(1-{\widehat{P}}_{i2}\right)\widehat{P_{i3}}\left(1-\widehat{P_{i4}}\right)\right]}}{{\displaystyle \sum_{i=1}^n\left[\left(1-\widehat{P_{i1}}\right)\widehat{P_{i2}}\widehat{P_{i3}}\left(1-\widehat{P_{i4}}\right)+\widehat{P_{i1}}\left(1-{\widehat{P}}_{i2}\right){\widehat{P}}_{i3}\left(1-{\widehat{P}}_{i4}\right)\right]}} $$

where *P*_*1*_, *P*_*2*_, *P*_*3*_ and *P*_*4*_ are the four taxa of the comparison and ${\hat{P}}_{ij}$ is the observed frequency of SNP *i* in population *j*. For whole genome estimation, we assigned scaffolds to 21 chromosomes according to *H. melpomene* linkage mapping and selected a block size of 50 kb, greater than the estimated linkage disequilibrium in *Heliconius*, to calculate the standard errors on *D*-statistics across 21 chromosomes [16]. An R package, bootstrap 2012-04, was used to perform the leave-one-out jackknife approach [72]. To identify candidate introgression loci, a small fixed window size (5 kb, 10 kb and 50 kb) was chosen for both *D*-statistic and the modified *f*-statistic (*f*_d_). *f*_d_ was calculated as [46]:

2 $$ \widehat{f}=\frac{S\left({P}_1,{P}_2,{P}_3,O\right)}{S\left({P}_1,{P}_D,{P}_D,O\right)} $$

where *P*_*1*_, *P*_*2*_, *P*_*3*_ and *O* are the four taxa of the comparison and *P*_*D*_ can be either *P*_*2*_ or *P*_*3*_, which has the higher frequency of the derived allele. For each 5 kb, 10 kb or 50 kb window, the standard error was calculated using a moving block bootstrap approach with the optimal block size being equal to *n*^1/3^, where *n* is the total number of values. Then all the tests were followed by two tailed z-tests to determine if the standard error for each *D* or *f* value was significantly different from zero, which indicates potential gene flow. For some of the candidate introgression loci, we also used the *D*-statistic to examine the potential of introgression between *H. m. nanna* and other silvaniform species, including *H. ethilla*, *H. ismenius*, *H. hecale* and *H. pardalinus* (Table S7 in Additional file 2)*.* Because we tested all 97 candidate introgression loci with the *D* and *f*_d_ statistics (and d_xy_, see below), we controlled for multiple testing using the Benjamini-Hochberg false discovery rate (FDR) method [73]. We used an FDR of 0.01 for all three tests, yielding adjusted significance thresholds of *P* < 0.006289 for *D*, *P* <0.007216 for *f*_d_, and *P* < 0.009381 for d_xy_.

### Population genetic analyses and sequencing depth estimation

To rule out false positive introgression loci due to incomplete lineage sorting, mean pairwise sequence divergence (d_xy_) was calculated among *H. m. nanna*, *H. besckei* and *H. numata* as a complementary analysis to the *D*-statistic and modified *f*-statistic [47]. Four individuals of the same species were used to calculate mean d_xy_ for each chromosome using a block size of 50 kb using the following equation [47]:

3 $$ {d}_{xy}=\frac{1}{n}{\displaystyle \sum_{i=1}^n\widehat{p_{ix}}\left(1-\widehat{p_{iy}}\right)}+\widehat{p_{iy}}\left(1-\widehat{p_{ix}}\right) $$

where *p*_*x*_ and *p*_*y*_ refer to reference allele frequency in taxon x and y. Then, standard error was calculated for each chromosome using a jackknife approach. For each candidate introgression locus, d_xy_ was calculated using a smaller block size of 100 bp to allow moving block bootstrapping. If the mean d_xy_ value of a putatively introgressed locus was lower than the mean value of its chromosome, the two values were compared statistically using a Mann-Whitney U-test. Sequencing depth and F_ST_ values of 50 kb adjacent windows were also calculated for the 21 chromosomes and candidate loci using VCFtools package [74].

### Annotating candidate introgressed loci

We extracted coding sequence regions for each candidate locus based on the genome annotation of *H. melpomene* [16] and performed NCBI-BLASTX against the nr database to characterize them.

### Ethical approval

No ethical approval was required.

### Availability of supporting data

Illumina paired-end whole genome resequencing data are available from NCBI Sequence Read Archive (SRA); all accession numbers are in Table S1 in Additional file 2. The *H. melpomene* reference genome, including annotation of version 1.1, is available at the *Heliconius* Genome Project website, http://www.butterflygenome.org/, as well as at Lepbase, http://lepbase.org/.

## Additional files

Additional file 1: Figure S1. A maximum-likelihood phylogeny of 32 samples combined with dating of the nodes. Divergence times were calibrated using a fast Bayesian approach based on the separation time of *melpomene* and *cydno-timareta* clades (1.3 ± 0.1 Mya) and the separation time of *H. pachinus* from the *cydno* clade (0.4 ± 0.1 Mya). Samples from *H. besckei* and *H. m. nanna* are highlighted in *red*. Numbers are in million years and *blue bars* stand for 95 % confidence intervals. **Figures S2-S9.** Maximum-likelihood phylogenetic trees were constructed for all candidate introgression loci using original windows (5 kb, 10 kb and 50 kb), expanded windows (original 5 kb + 5 kb on either side, original 10 kb + 10 kb on either side) and multiple 10 kb windows within original 50 kb windows. The window size (as shown in each figure) may vary due to actual scaffold length and position. **Figure S10.** A maximum-likelihood phylogeny of *Heliconius* butterflies based on the SNP data from the Z chromosome. The *cydno*-*timareta*-*melpomene* clade is grouped with a subset of silvaniform species (highlighted in *blue*). (PDF 715 kb) Additional file 2: Table S1. Sample information and sequencing statistics. **Table S2.** Results of chromosomal *D*-statistics. **Table S3.** *D*-statistics and *f*_*d*_-statistics for all the predicted candidate introgression loci. **Table S4.** Results of chromosomal d_xy_. **Table S5.** Results of d_xy_ for all the candidate introgression loci. **Table S6.** Read depth analyses for all the predicted candidate introgression loci among four focal species. **Table S7.** Results of *D*-statistics to examine gene flow between other species and *H. m. nanna*. **Table S8.** Gene annotations of 41 candidate introgression loci. **Table S9.** Autosome versus Z chromosome population genetic statistics. (DOCX 123 kb)

## Abbreviations

d_xy_: mean pairwise sequence divergence
FDR: False discovery rate
Mya: Million years ago
SNP: Single nucleotide polymorphism
