# Supplementary material for: Genome-wide introgression among distantly related Heliconius butterfly species
Source: Genome Biol. 2016 Feb 27;17:25. doi: 10.1186/s13059-016-0889-0 (PMC4769579; doi:10.1186/s13059-016-0889-0)
Supplement: Additional file 1: Figure S1. — A maximum-likelihood phylogeny of 32 samples combined with dating of the nodes. Divergence times were calibrated using a fast Bayesian approach based on the separation time of melpomene and cydno-timareta clades (1.3 ± 0.1 Mya) and the separation time of H. pachinus from the cydno clade (0.4 ± 0.1 Mya). Samples from H. besckei and H. m. nanna are highlighted in red. Numbers are in million years and blue bars stand for 95 % confidence intervals. Figures S2-S9. Maximum-likelihood phylogenetic trees were constructed for all candidate introgression loci using original windows (5 kb, 10 kb and 50 kb), expanded windows (original 5 kb + 5 kb on either side, original 10 kb + 10 kb on either side) and multiple 10 kb windows within original 50 kb windows. The window size (as shown in each figure) may vary due to actual scaffold length and position. Figure S10. A maximum-likelihood phylogeny of Heliconius butterflies based on the SNP data from the Z chromosome. The cydno-timareta-melpomene clade is grouped with a subset of silvaniform species (highlighted in blue). (PDF 715 kb) [file 13059_2016_889_MOESM1_ESM.pdf]

Figure S1

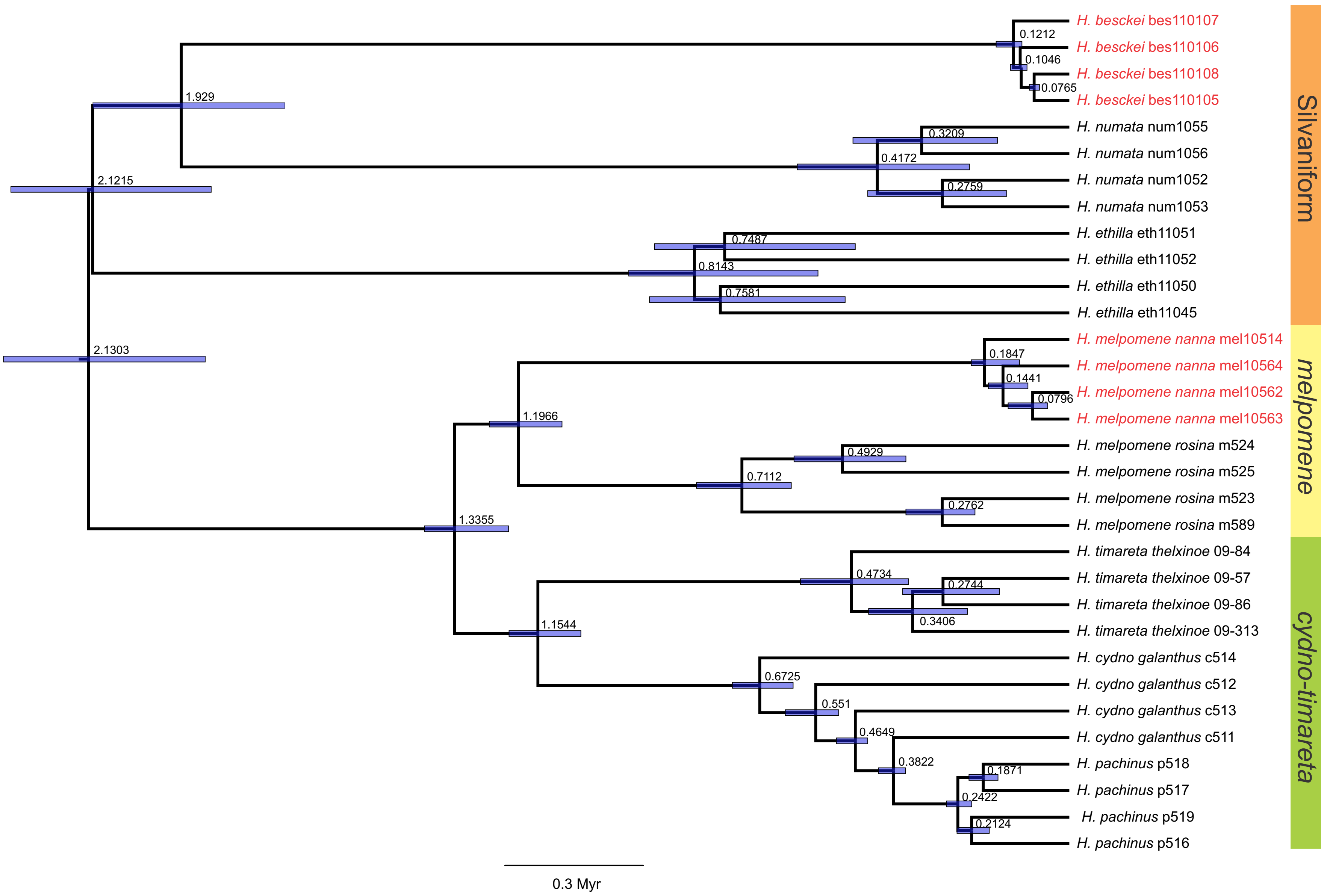

H. besckei bes110107
H. besckei bes110106
H. besckei bes110108
H. besckei bes110105
H. numata num1055
H. numata num1056
H. numata num1052
H. numata num1053
H. ethilla eth11051
H. ethilla eth11052
H. ethilla eth11050
H. ethilla eth11045
H. melpomene nanna mel10514
H. melpomene nanna mel10564
H. melpomene nanna mel10562
H. melpomene nanna mel10563
H. melpomene rosina m524
H. melpomene rosina m525
H. melpomene rosina m523
H. melpomene rosina m589
H. timareta thelxinoe 09-84
H. timareta thelxinoe 09-57
H. timareta thelxinoe 09-86
H. timareta thelxinoe 09-313
H. cydno galanthus c514
H. cydno galanthus c512
H. cydno galanthus c513
H. cydno galanthus c511
H. pachinus p518
H. pachinus p517
H. pachinus p519
H. pachinus p516
Silvaniform
melpomene
cydno-timareta
1.929
2.1215
2.1303
0.1212
0.1046
0.0765
0.3209
0.4172
0.2759
0.7487
0.8143
0.7581
0.1847
0.1441
0.0796
1.1966
0.4929
0.7112
0.2762
1.3355
0.4734
0.2744
0.3406
1.1544
0.6725
0.551
0.4649
0.3822
0.1871
0.2422
0.2124
0.3 Myr

Figure S2

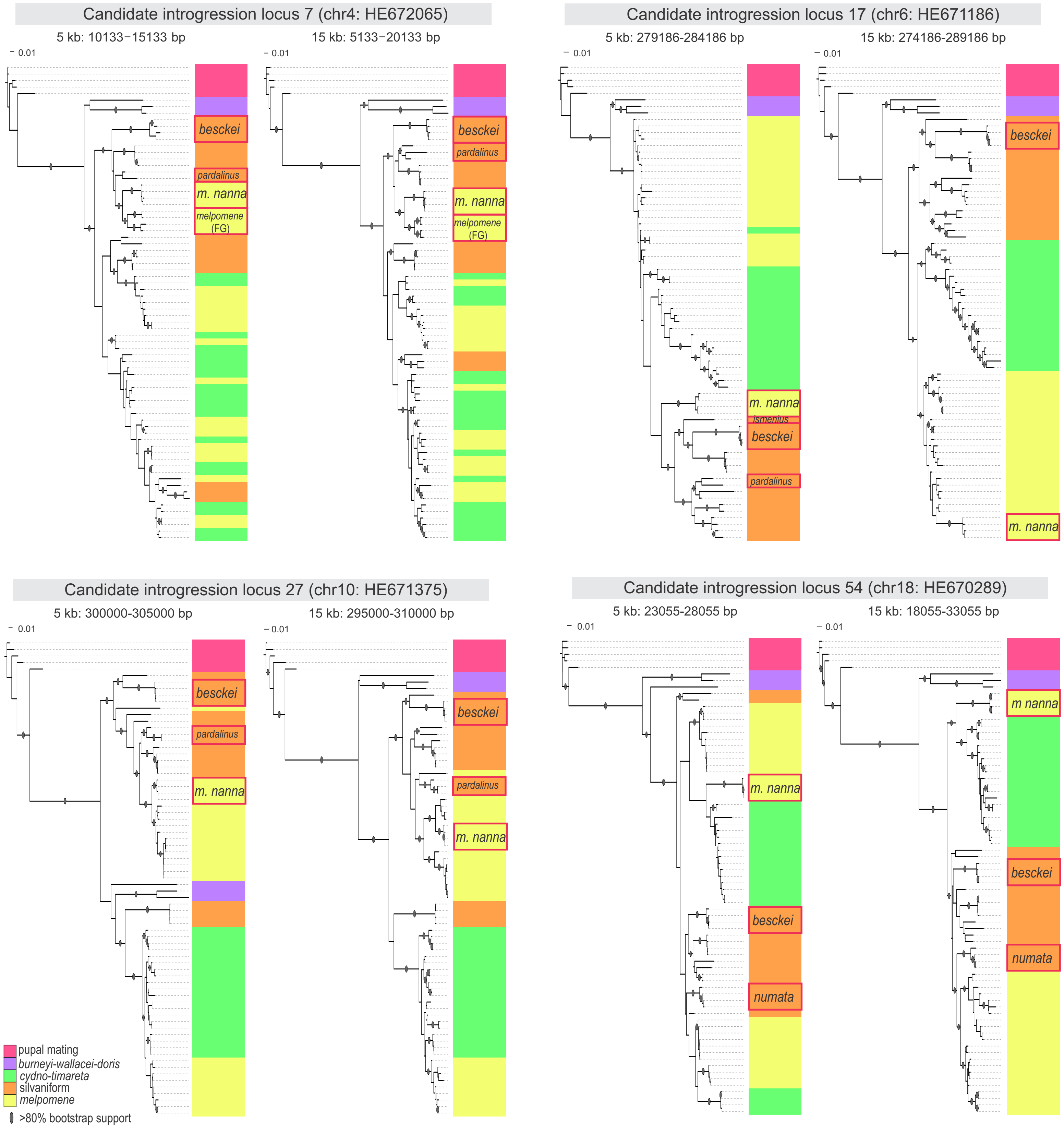
Candidate introgression locus 7 (chr4: HE672065)
5 kb: 10133–15133 bp
15 kb: 5133–20133 bp
0.01
besckei
pardalinus
m. nanna
melpomene (FG)
Candidate introgression locus 17 (chr6: HE671186)
5 kb: 279186-284186 bp
15 kb: 274186-289186 bp
m. nanna
ismenius
besckei
pardalinus
Candidate introgression locus 27 (chr10: HE671375)
5 kb: 300000-305000 bp
15 kb: 295000-310000 bp
Candidate introgression locus 54 (chr18: HE670289)
5 kb: 23055-28055 bp
15 kb: 18055-33055 bp
m nanna
numata
pupal mating
burneyi-wallacei-doris
cydno-timareta
silvaniform
melpomene
>80% bootstrap support

Figure S3

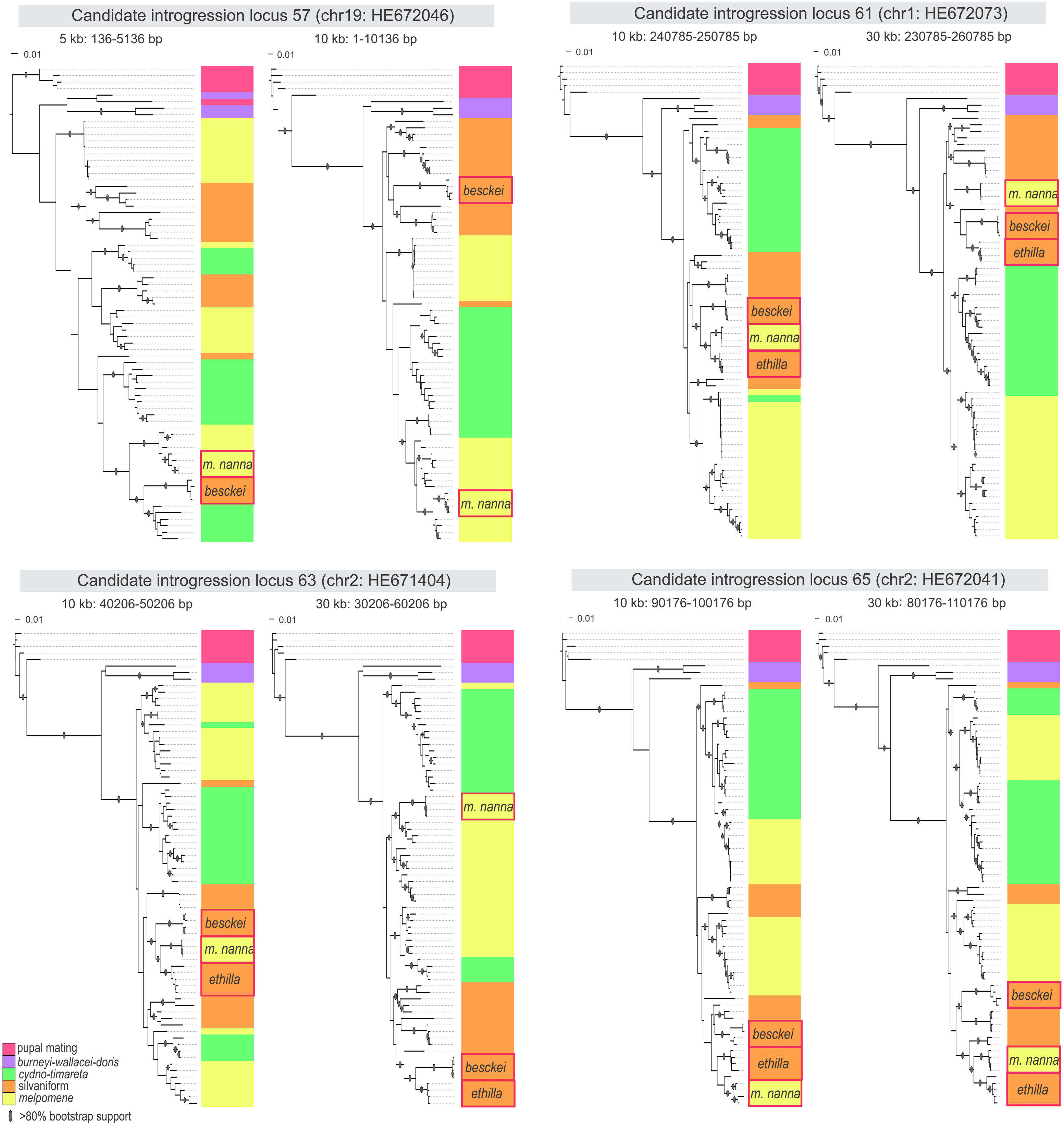
Candidate introgression locus 57 (chr19: HE672046)
5 kb: 136-5136 bp
10 kb: 1-10136 bp
Candidate introgression locus 61 (chr1: HE672073)
10 kb: 240785-250785 bp
30 kb: 230785-260785 bp
Candidate introgression locus 63 (chr2: HE671404)
10 kb: 40206-50206 bp
30 kb: 30206-60206 bp
Candidate introgression locus 65 (chr2: HE672041)
10 kb: 90176-100176 bp
30 kb: 80176-110176 bp
0.01
m. nanna
besckei
ethilla
pupal mating
burneyi-wallacei-doris
cydno-timareta
silvaniform
melpomene
>80% bootstrap support

Figure S4

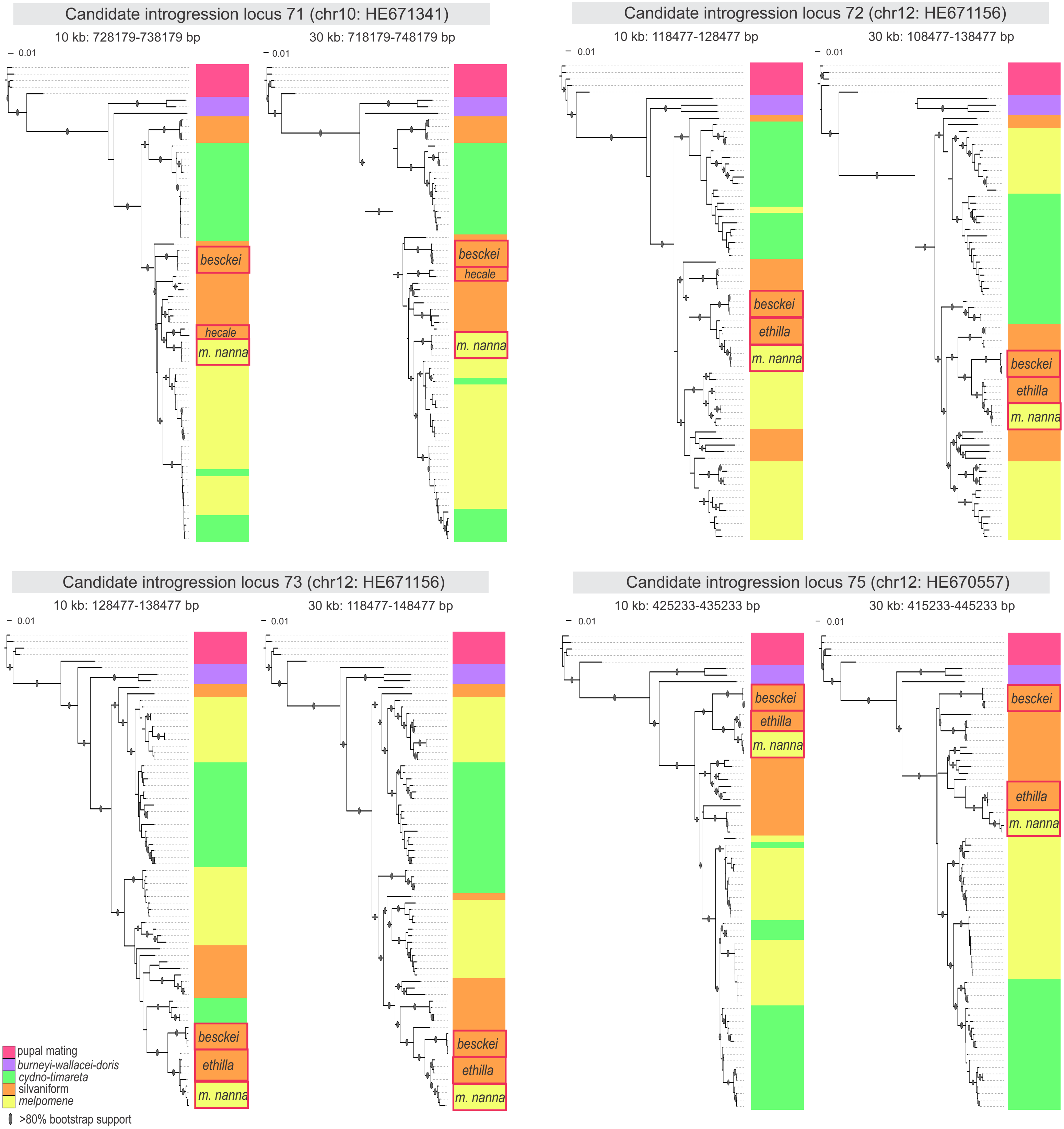
Candidate introgression locus 71 (chr10: HE671341)
10 kb: 728179-738179 bp
30 kb: 718179-748179 bp
0.01
besckei
hecale
m. nanna
Candidate introgression locus 72 (chr12: HE671156)
10 kb: 118477-128477 bp
30 kb: 108477-138477 bp
besckei
ethilla
m. nanna
Candidate introgression locus 73 (chr12: HE671156)
10 kb: 128477-138477 bp
30 kb: 118477-148477 bp
besckei
ethilla
m. nanna
Candidate introgression locus 75 (chr12: HE670557)
10 kb: 425233-435233 bp
30 kb: 415233-445233 bp
besckei
ethilla
m. nanna
pupal mating
burneyi-wallacei-doris
cydno-timareta
silvaniform
melpomene
>80% bootstrap support

Figure S5

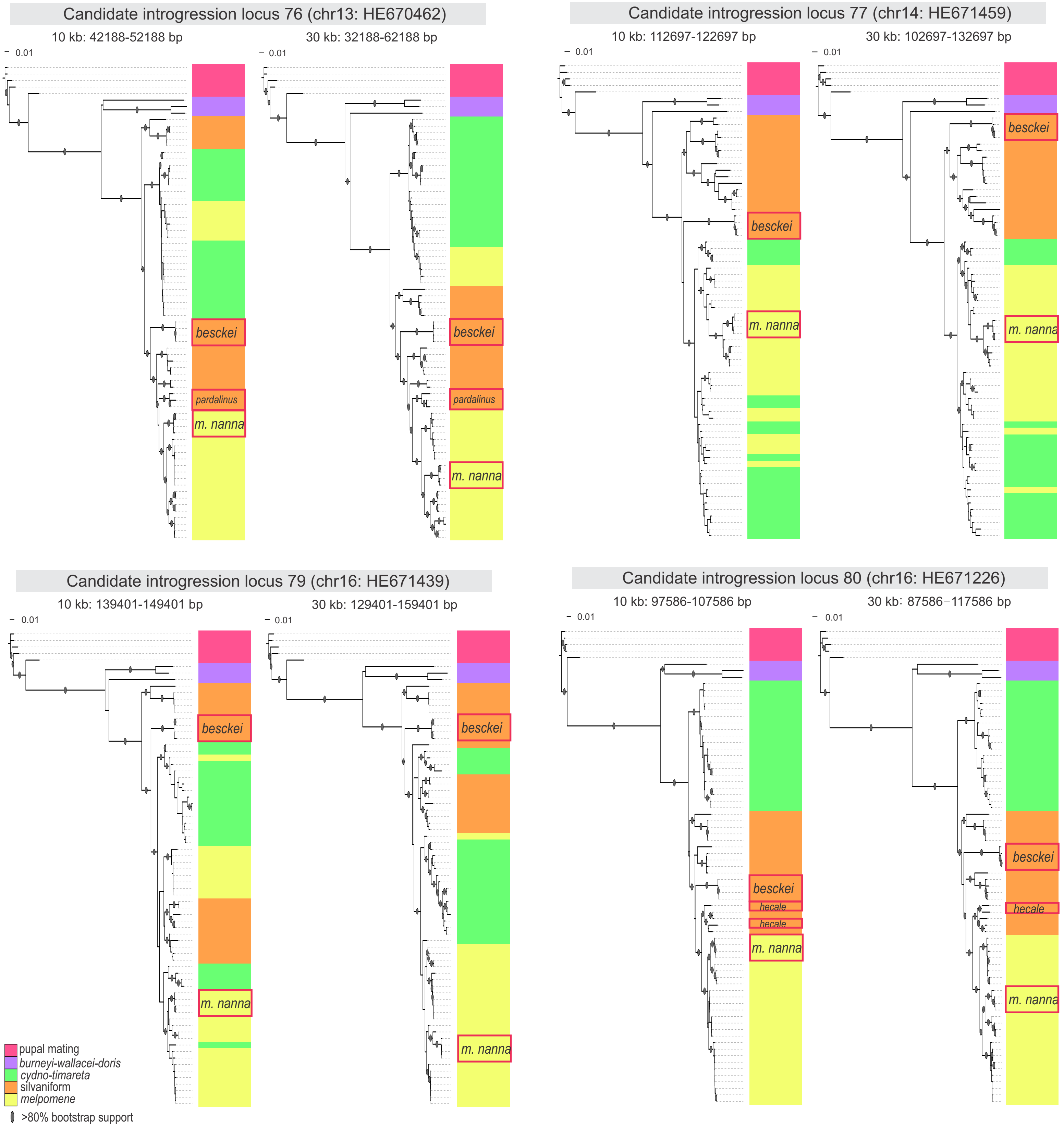

Candidate introgression locus 76 (chr13: HE670462)
10 kb: 42188-52188 bp
30 kb: 32188-62188 bp
0.01
besckei
pardalinus
m. nanna
Candidate introgression locus 77 (chr14: HE671459)
10 kb: 112697-122697 bp
30 kb: 102697-132697 bp
0.01
besckei
m. nanna
Candidate introgression locus 79 (chr16: HE671439)
10 kb: 139401-149401 bp
30 kb: 129401-159401 bp
0.01
besckei
m. nanna
Candidate introgression locus 80 (chr16: HE671226)
10 kb: 97586-107586 bp
30 kb: 87586−117586 bp
0.01
besckei
hecale
m. nanna
pupal mating
burneyi-wallacei-doris
cydno-timareta
silvaniform
melpomene
>80% bootstrap support

Figure S6

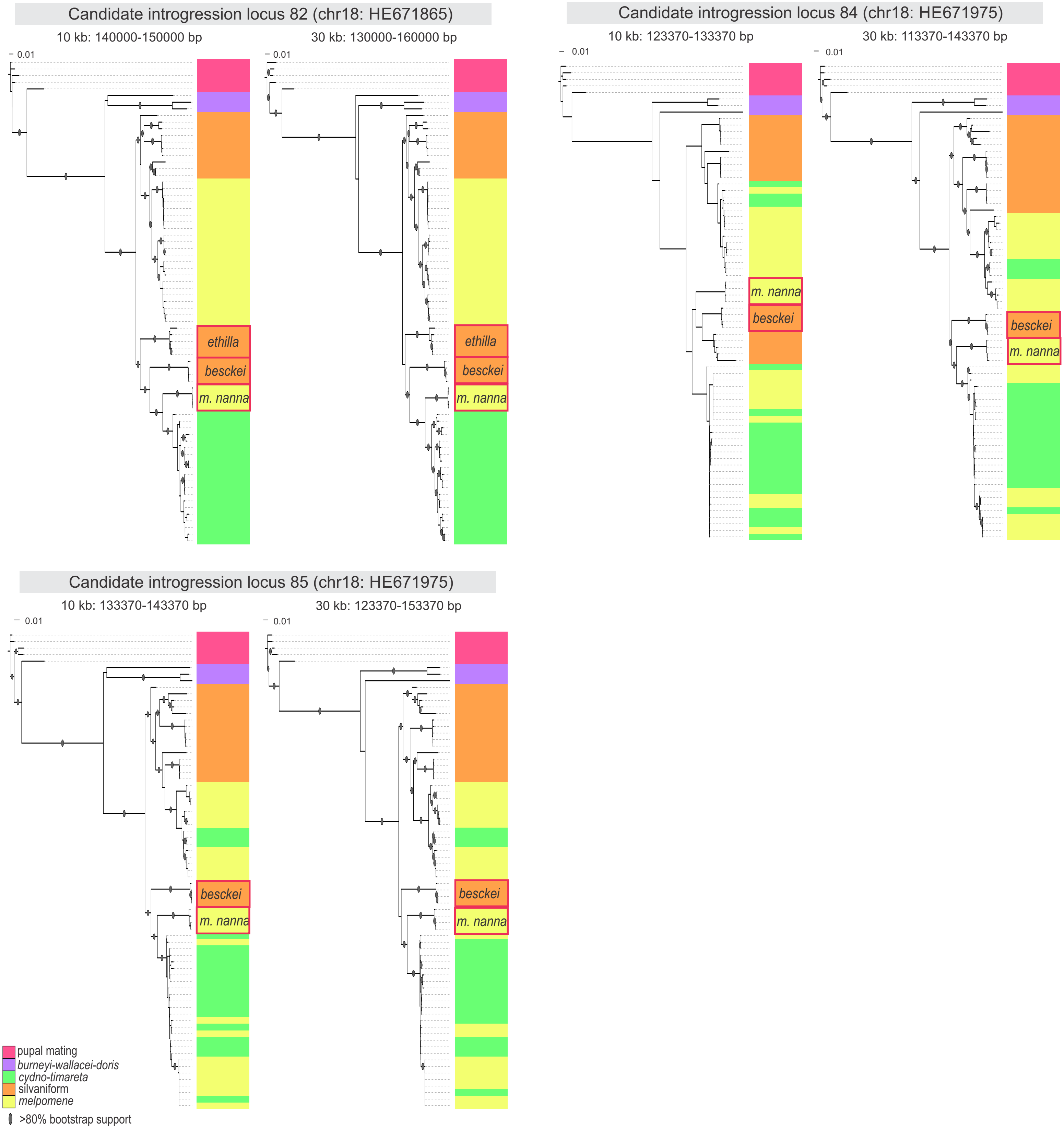

Candidate introgression locus 82 (chr18: HE671865)
10 kb: 140000-150000 bp
30 kb: 130000-160000 bp
0.01
ethilla
besckei
m. nanna
Candidate introgression locus 84 (chr18: HE671975)
10 kb: 123370-133370 bp
30 kb: 113370-143370 bp
m. nanna
besckei
Candidate introgression locus 85 (chr18: HE671975)
10 kb: 133370-143370 bp
30 kb: 123370-153370 bp
besckei
m. nanna
pupal mating
burneyi-wallacei-doris
cydno-timareta
silvaniform
melpomene
>80% bootstrap support

Figure S7

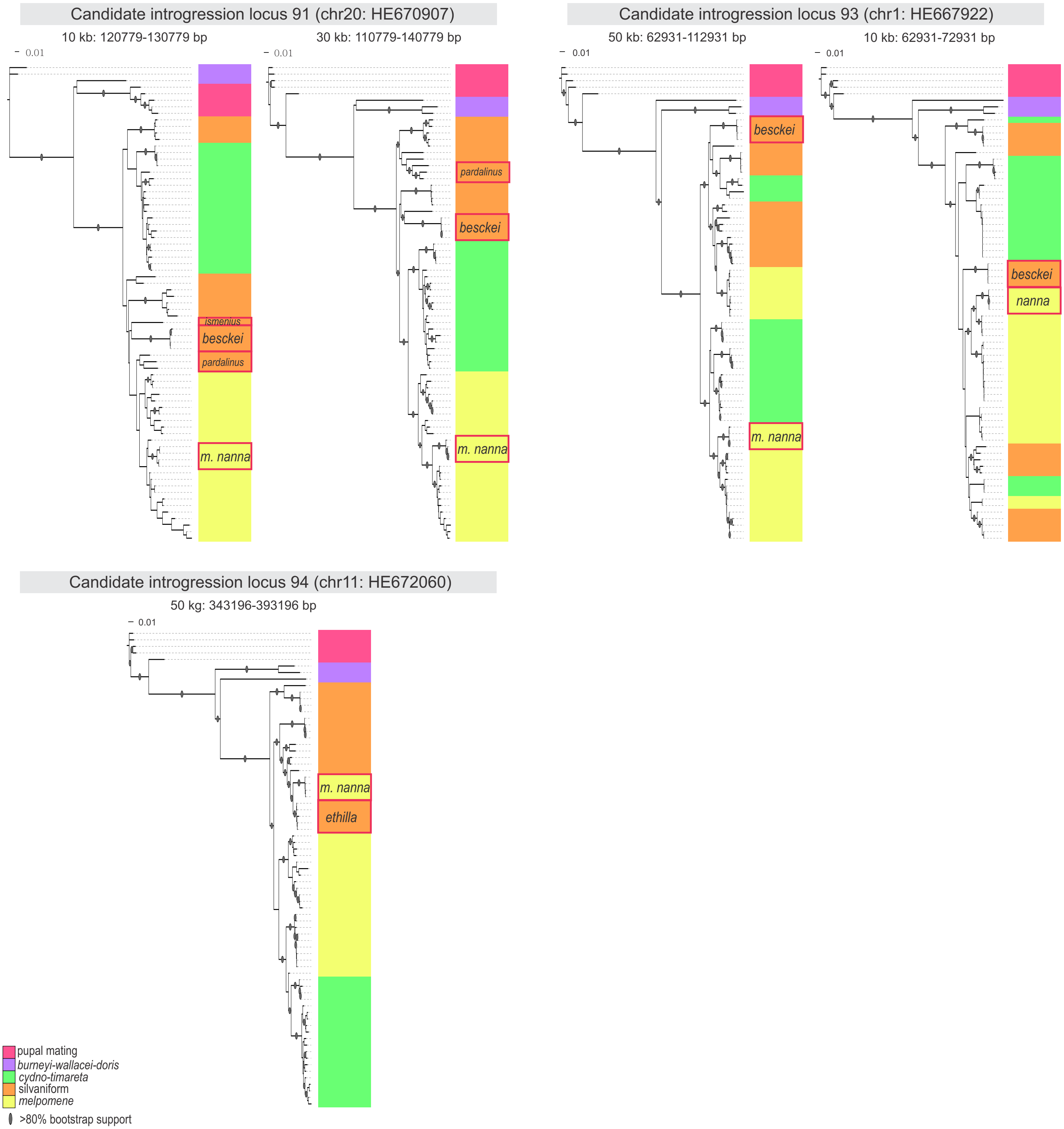
Candidate introgression locus 91 (chr20: HE670907)
10 kb: 120779-130779 bp
30 kb: 110779-140779 bp
0.01
ismenius
besckei
pardalinus
m. nanna
Candidate introgression locus 93 (chr1: HE667922)
50 kb: 62931-112931 bp
10 kb: 62931-72931 bp
besckei
nanna
Candidate introgression locus 94 (chr11: HE672060)
50 kg: 343196-393196 bp
ethilla
pupal mating
burneyi-wallacei-doris
cydno-timareta
silvaniform
melpomene
>80% bootstrap support

Figure S8

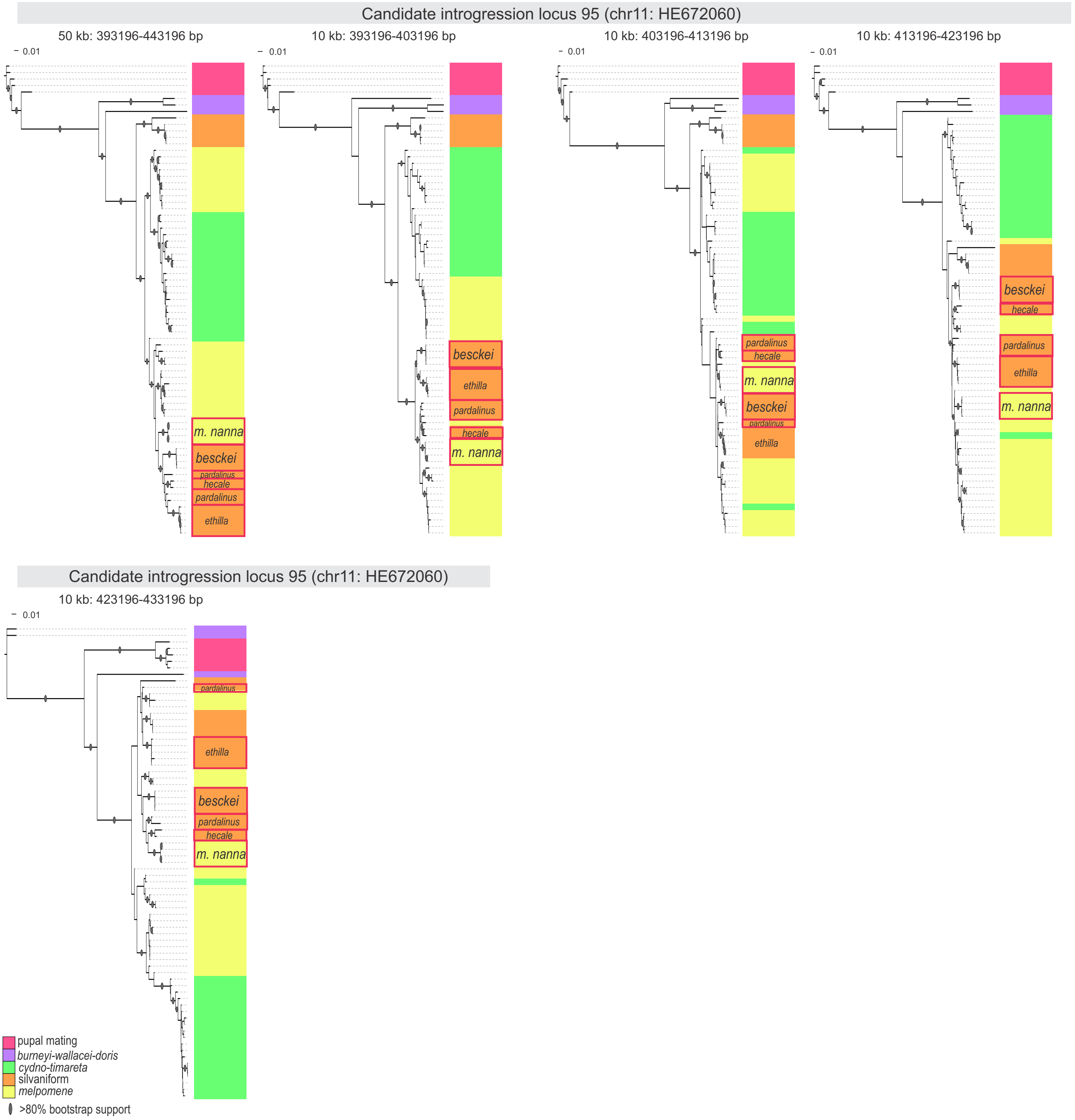

Candidate introgression locus 95 (chr11: HE672060)
50 kb: 393196-443196 bp
10 kb: 393196-403196 bp
10 kb: 403196-413196 bp
10 kb: 413196-423196 bp
0.01
m. nanna
besckei
pardalinus
hecale
pardalinus
ethilla
besckei
ethilla
pardalinus
hecale
m. nanna
pardalinus
hecale
m. nanna
besckei
pardalinus
ethilla
besckei
hecale
pardalinus
ethilla
m. nanna
Candidate introgression locus 95 (chr11: HE672060)
10 kb: 423196-433196 bp
pardalinus
ethilla
besckei
pardalinus
hecale
m. nanna
pupal mating
burneyi-wallacei-doris
cydno-timareta
silvaniform
melpomene
>80% bootstrap support

Figure S9

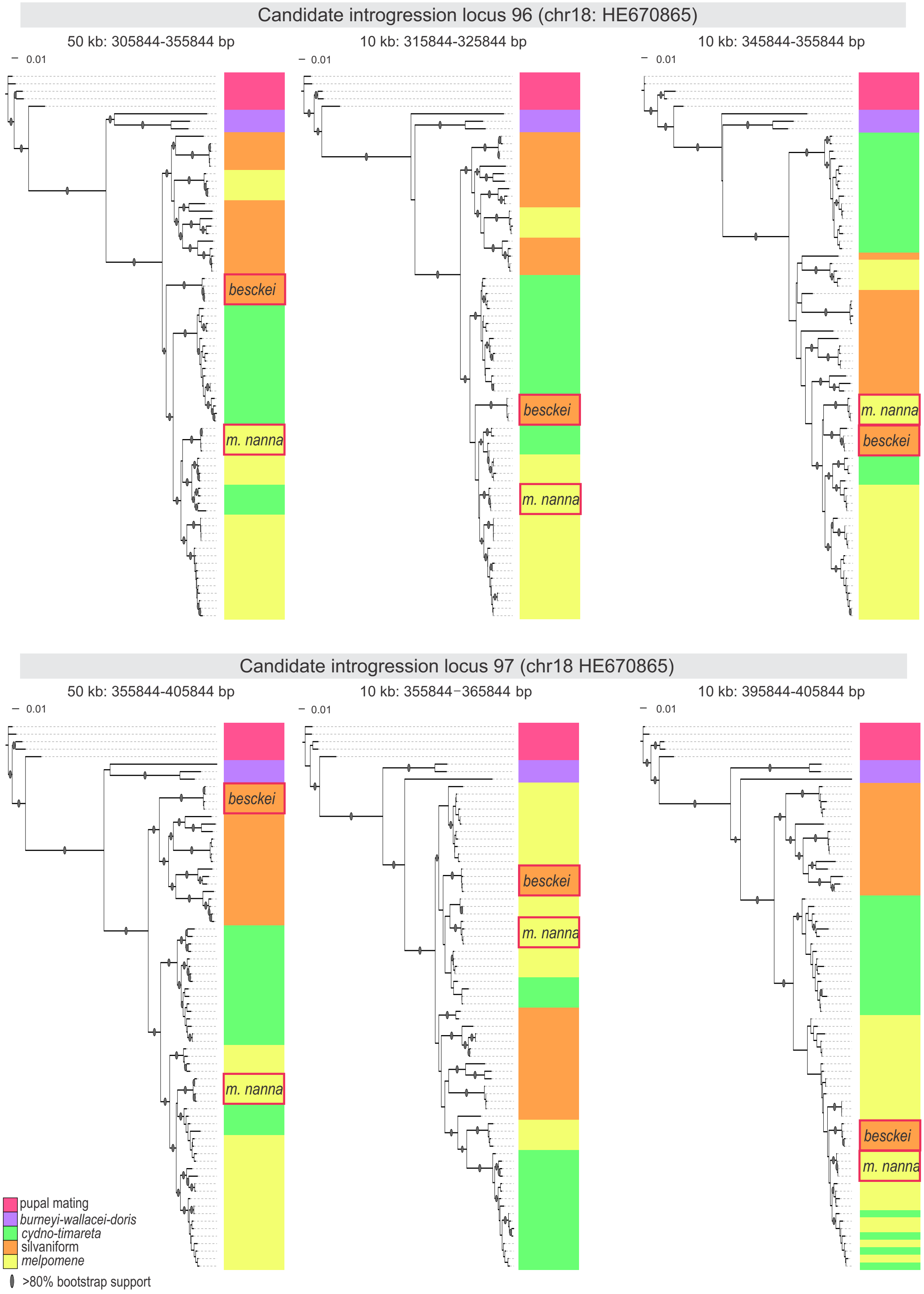
Candidate introgression locus 96 (chr18: HE670865)
50 kb: 305844-355844 bp
10 kb: 315844-325844 bp
10 kb: 345844-355844 bp
0.01
besckei
m. nanna
Candidate introgression locus 97 (chr18 HE670865)
50 kb: 355844-405844 bp
10 kb: 355844−365844 bp
10 kb: 395844-405844 bp
pupal mating
burneyi-wallacei-doris
cydno-timareta
silvaniform
melpomene
>80% bootstrap support

Figure S10

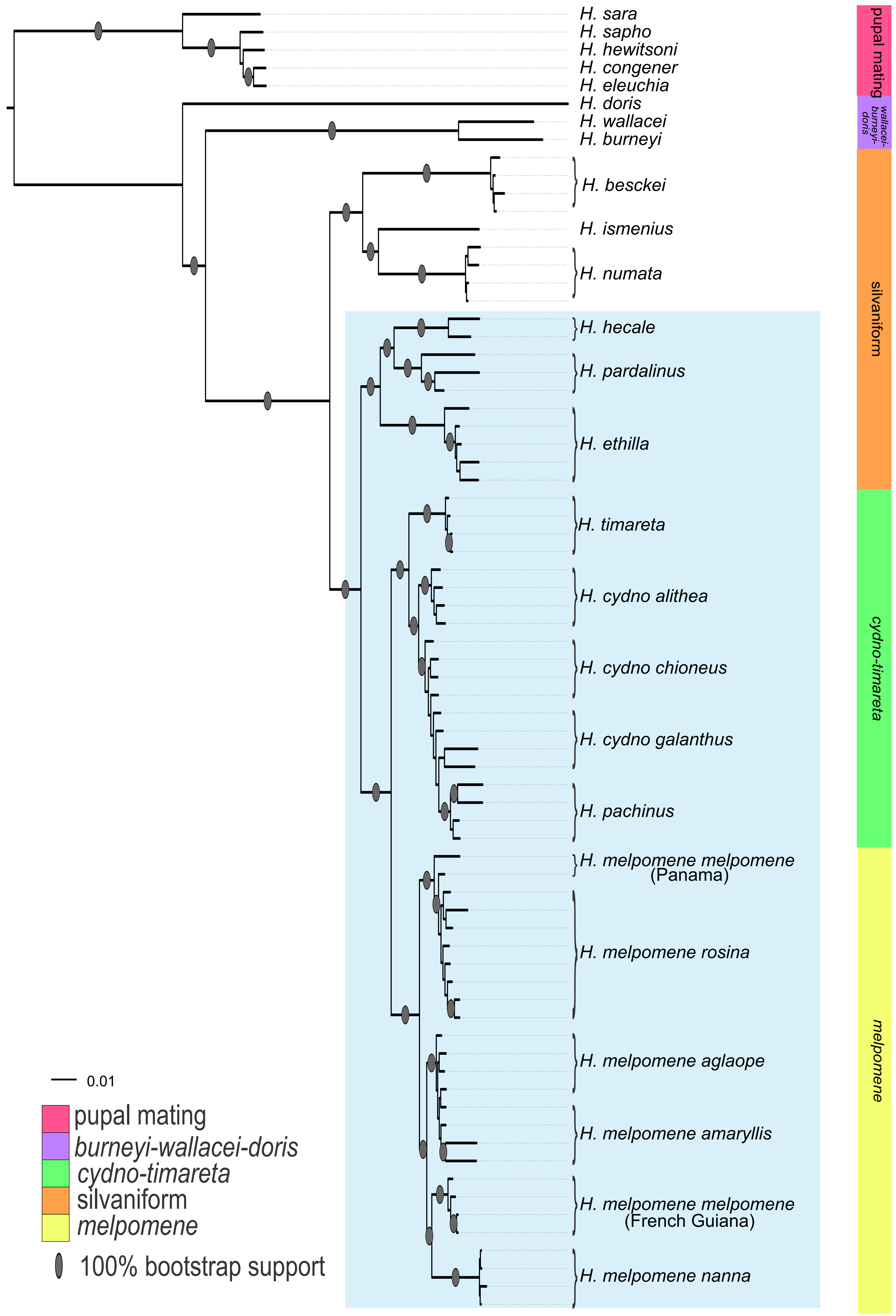

H. sara
H. sapho
H. hewitsoni
H. congener
H. eleuchia
H. doris
H. wallacei
H. burneyi
H. besckei
H. ismenius
H. numata
H. hecale
H. pardalinus
H. ethilla
H. timareta
H. cydno alithea
H. cydno chioneus
H. cydno galanthus
H. pachinus
H. melpomene melpomene (Panama)
H. melpomene rosina
H. melpomene aglaope
H. melpomene amaryllis
H. melpomene melpomene (French Guiana)
H. melpomene nanna
pupal mating
wallacei-burneyi-doris
silvaniform
cydno-timareta
melpomene
0.01
pupal mating
burneyi-wallacei-doris
cydno-timareta
silvaniform
melpomene
100% bootstrap support
