# Supplementary material for: Genome-wide introgression among distantly related Heliconius butterfly species
Source: Genome Biol. 2016 Feb 27;17:25. doi: 10.1186/s13059-016-0889-0 (PMC4769579; doi:10.1186/s13059-016-0889-0)
Supplement: Additional file 2: Table S1. — Sample information and sequencing statistics. Table S2. Results of chromosomal D-statistics. Table S3. D-statistics and f d-statistics for all the predicted candidate introgression loci. Table S4. Results of chromosomal dxy. Table S5. Results of dxy for all the candidate introgression loci. Table S6. Read depth analyses for all the predicted candidate introgression loci among four focal species. Table S7. Results of D-statistics to examine gene flow between other species and H. m. nanna. Table S8. Gene annotations of 41 candidate introgression loci. Table S9. Autosome versus Z chromosome population genetic statistics. (DOCX 123 kb) [file 13059_2016_889_MOESM2_ESM.docx]

**Table S1.** Sample information and sequencing statistics.

| **Sample** | **Taxon** | | **Country** | **Data source** | **Reads (Gb)** | **Genotype calls (Qual>30)** | **Mean Depth (Qual>30)** |
| --- | --- | --- | --- | --- | --- | --- | --- |
|  |  |  |  |  |  |  |  |
| c511 | *H. cydno galanthus* | | Costa Rica | NCBI PRJNA226620 | 5.26 | 88210173 | 14.7383 |
| c512 | *H. cydno galanthus* | | Costa Rica | NCBI PRJNA226620 | 5.31 | 88244559 | 14.8701 |
| c513 | *H. cydno galanthus* | | Costa Rica | NCBI PRJNA226620 | 5.33 | 88079532 | 15.2309 |
| c514 | *H. cydno galanthus* | | Costa Rica | NCBI PRJNA226620 | 5.31 | 87986423 | 14.9557 |
| p516 | *H. pachinus* | | Costa Rica | NCBI PRJNA226620 | 5.48 | 87395367 | 15.1856 |
| p517 | *H. pachinus* | | Costa Rica | NCBI PRJNA226620 | 5.48 | 87416354 | 15.5574 |
| p518 | *H. pachinus* | | Costa Rica | NCBI PRJNA226620 | 5.48 | 87413374 | 15.6924 |
| p519 | *H. pachinus* | | Costa Rica | NCBI PRJNA226620 | 5.47 | 87312140 | 15.3332 |
| h665 | *H. hecale* | | Costa Rica | NCBI PRJNA226620 | 5.28 | 84484185 | 12.8741 |
| i02_210 | *H. ismenius* | | Costa Rica | NCBI PRJNA226620 | 5.23 | 80803979 | 11.7234 |
| m523 | *H. melpomene rosina* | | Costa Rica | NCBI PRJNA226620 | 5.35 | 89593520 | 15.6653 |
| m524 | *H. melpomene rosina* | | Costa Rica | NCBI PRJNA226620 | 5.29 | 89754094 | 15.5988 |
| m525 | *H. melpomene rosina* | | Costa Rica | NCBI PRJNA226620 | 5.33 | 90059481 | 15.2536 |
| m589 | *H. melpomene rosina* | | Costa Rica | NCBI PRJNA226620 | 5.30 | 89485066 | 15.2457 |
| mACw172 | *H. cydno alithea* | | Ecuador | NCBI PRJNA308754 | 4.45 | 85844325 | 11.3393 |
| mACy20 | *H. cydno alithea* | | Ecuador | NCBI PRJNA308754 | 3.77 | 86580959 | 10.2762 |
| nACy112 | *H. cydno alithea* | | Ecuador | NCBI PRJNA308754 | 4.82 | 86700310 | 12.0696 |
| nACw212 | *H. cydno alithea* | | Ecuador | NCBI PRJNA308754 | 4.38 | 84955389 | 10.1039 |
| 553 | *H. cydno chioneus* | | Panama | ENA ERP002440 | 11.39 | 88099546 | 32.7726 |
| 560 | *H. cydno chioneus* | | Panama | ENA ERP002440 | 11.11 | 88107962 | 32.3095 |
| 564 | *H. cydno chioneus* | | Panama | ENA ERP002440 | 12.27 | 88243940 | 35.6369 |
| 565 | *H. cydno chioneus* | | Panama | ENA ERP002440 | 15.00 | 88564014 | 42.4312 |
| 531 | *H. melpomene rosina* | | Panama | ENA ERP002440 | 8.18 | 88437616 | 24.7658 |
| 533 | *H. melpomene rosina* | | Panama | ENA ERP002440 | 8.23 | 87736915 | 24.5514 |
| 546 | *H. melpomene rosina* | | Panama | ENA ERP002440 | 8.10 | 87854008 | 24.2645 |
| 2071 | *H. melpomene rosina* | | Panama | ENA ERP002440 | 11.16 | 90001903 | 34.2321 |
| 18038 | *H. melpomene melpomene* | | Panama | ENA ERP002440 | 20.53 | 90318963 | 53.3984 |
| 18097 | *H. melpomene melpomene* | Panama | | ENA ERP002440 | 5.04 | 87141883 | 13.6327 |
| 9315 | *H. melpomene melpomene* | French Guiana | | ENA ERP002440 | 9.19 | 87333202 | 21.232 |
| 9316 | *H. melpomene melpomene* | French Guiana | | ENA ERP002440 | 8.61 | 87003485 | 20.1798 |
| 9317 | *H. melpomene melpomene* | French Guiana | | ENA ERP002440 | 11.12 | 87933247 | 30.7822 |
| 13435 | *H. melpomene melpomene* | French Guiana | | ENA ERP002440 | 11.36 | 87984945 | 31.6378 |
| 11-48 | *H. melpomene amaryllis* | Peru | | ENA ERP002440 | 18.93 | 89767573 | 50.2608 |
| 11-160 | *H. melpomene amaryllis* | Peru | | ENA ERP002440 | 14.97 | 89494839 | 39.3362 |
| 09-216 | *H. melpomene amaryllis* | Peru | | ENA ERP002440 | 11.16 | 89444316 | 28.5186 |
| 11-293 | *H. melpomene amaryllis* | Peru | | ENA ERP002440 | 18.36 | 89641231 | 48.0002 |
| 09-108 | *H. melpomene aglaope* | Peru | | ENA ERP002440 | 14.15 | 89348115 | 30.8067 |
| 09-112 | *H. melpomene aglaope* | Peru | | ENA ERP002440 | 13.17 | 89611000 | 34.7355 |
| 11-569 | *H. melpomene aglaope* | Peru | | ENA ERP002440 | 15.32 | 89716235 | 39.2725 |
| 11-572 | *H. melpomene aglaope* | Peru | | ENA ERP002440 | 12.94 | 89543327 | 32.5363 |
| 09-57 | *H. timareta thelxinoe* | Peru | | ENA ERP002440 | 19.30 | 88998694 | 39.1815 |
| 09-84 | *H. timareta thelxinoe* | Peru | | ENA ERP002440 | 11.21 | 88737873 | 27.1564 |
| 09-86 | *H. timareta thelxinoe* | Peru | | ENA ERP002440 | 13.54 | 89113345 | 35.9559 |
| 09-313 | *H. timareta thelxinoe* | Peru | | ENA ERP002440 | 12.04 | 88849374 | 32.231 |
| 09-371 | *H. pardalinus ssp. nov.* | Peru | | ENA ERP002440 | 12.28 | 88227077 | 29.3205 |
| 09-202 | *H. pardalinus sergestus* | Peru | | ENA ERP002440 | 15.01 | 87709237 | 33.427 |
| 09-67 | *H. ethilla aerotome* | Peru | | ENA ERP002440 | 15.97 | 85682193 | 38.8418 |
| 09-273 | *H. hecale felix* | Peru | | ENA ERP002440 | 16.27 | 87071441 | 35.2363 |
| mel10514 | *H. melpomene nanna* | Brazil | | NCBI PRJNA308754 | 5.39 | 85793027 | 14.3636 |
| mel10562 | *H. melpomene nanna* | Brazil | | NCBI PRJNA308754 | 5.36 | 85879844 | 14.7871 |
| mel10563 | *H. melpomene nanna* | Brazil | | NCBI PRJNA308754 | 7.18 | 85771871 | 19.0891 |
| mel10564 | *H. melpomene nanna* | Brazil | | NCBI PRJNA308754 | 5.88 | 85775958 | 15.6235 |
| eth11045 | *H. ethilla narcaea* | Brazil | | NCBI PRJNA308754 | 7.41 | 84503245 | 19.6327 |
| eth11050 | *H. ethilla narcaea* | Brazil | | NCBI PRJNA308754 | 4.85 | 83432066 | 13.1736 |
| eth11051 | *H. ethilla narcaea* | Brazil | | NCBI PRJNA308754 | 4.99 | 83577550 | 13.2193 |
| eth11052 | *H. ethilla narcaea* | Brazil | | NCBI PRJNA308754 | 7.43 | 84213894 | 19.757 |
| num1052 | *H. numata robigus* | Brazil | | NCBI PRJNA308754 | 6.33 | 83209853 | 15.7755 |
| num1053 | *H. numata robigus* | Brazil | | NCBI PRJNA308754 | 7.87 | 84055769 | 19.5548 |
| num1055 | *H. numata robigus* | Brazil | | NCBI PRJNA308754 | 4.85 | 83034333 | 12.2432 |
| num1056 | *H. numata robigus* | Brazil | | NCBI PRJNA308754 | 4.69 | 82923668 | 12.0605 |
| bes110105 | *H. besckei* | Brazil | | NCBI PRJNA308754 | 5.12 | 80823972 | 12.0438 |
| bes110106 | *H. besckei* | Brazil | | NCBI PRJNA308754 | 5.12 | 80446455 | 12.2099 |
| bes110107 | *H. besckei* | Brazil | | NCBI PRJNA308754 | 8.49 | 81553238 | 20.2144 |
| bes110109 | *H. besckei* | Brazil | | NCBI PRJNA308754 | 5.71 | 80775790 | 13.5974 |
| eleu08001 | *H. eleuchia* | Ecuador | | NCBI PRJNA308754 | 11.74 | 50312651 | 10.7113 |
| con9125 | *H. congener* | Ecuador | | NCBI PRJNA308754 | 11.40 | 49738924 | 10.0649 |
| hew02130 | *H. hewitsoni* | Costa Rica | | NCBI PRJNA308754 | 11.68 | 50587773 | 11.7595 |
| saph08100 | *H. sapho candidus* | Ecuador | | NCBI PRJNA308754 | 11.50 | 51039752 | 11.2966 |
| sar08025 | *H. sara veraepacis* | Ecuador | | NCBI PRJNA308754 | 13.32 | 53290580 | 13.95 |
| par11835 | *H. pardalinus butleri* | Peru | | NCBI RJNA308754 | 10.42 | 87914488 | 24.9754 |
| Hel_doris | *H. doris* | Peru | | NCBI PRJNA308754 | 13.20 | 56347958 | 11.6819 |
| Hel_burneyi | *H. burneyi* | Peru | | NCBI PRJNA308754 | 12.08 | 60391524 | 12.7453 |
| Hel_wallacei | *H. wallacei* | Peru | | NCBI PRJNA308754 | 13.80 | 61576769 | 14.6008 |

**Table S2.** Results of chromosomal *D*-statistics.

|  | ***D* (*numata*, *besckei*, *m. nanna*, *wallacei*)** |
| --- | --- |
| chr1 | -0.0959±0.0052*** |
| chr2 | -0.0872±0.0125*** |
| chr3 | -0.1087±0.0066*** |
| chr4 | -0.1066±0.0064*** |
| chr5 | -0.1213±0.0074*** |
| chr6 | -0.1285±0.0069*** |
| chr7 | -0.1071±0.0068*** |
| chr8 | -0.1009±0.0074*** |
| chr9 | -0.1139±0.0059*** |
| chr10 | -0.0991±0.0047*** |
| chr11 | -0.1260±0.0090*** |
| chr12 | -0.0956±0.0052*** |
| chr13 | -0.1044±0.0057*** |
| chr14 | -0.1021±0.0084*** |
| chr15 | -0.0967±0.0082*** |
| chr16 | -0.1143±0.0064*** |
| chr17 | -0.1284±0.0066*** |
| chr18 | -0.0944±0.0056*** |
| chr19 | -0.0882±0.0054*** |
| chr20 | -0.1100±0.0088*** |
| chrZ | -0.0362±0.0087*** |

*** indicates p<0.001

**Table S3.** *D*-statistics and *f_d_*-statistics for all the candidate introgression loci.

| **ID** | **Chr** | **Scaf** | **length (kb)** | ***D*** | ***D* mean** | ***f_d_*** | ***f_d_* mean** |
| --- | --- | --- | --- | --- | --- | --- | --- |
| 1 | chr1:6425000-6430000 | HE669357:205937-210937 | 5 | 0.7961 | 0.6940±0.1791 | 0.2157 | 0.1774±0.0395 |
| 2^b^ | chr1:15495000-15500000 | HE671415:36614-41614 | 5 | 1.0000 | 1.0000±0.0000 | 0.0664 | 0.0584±0.0302 |
| 3^b^ | chr2:120000-125000 | HE671404:10206-15206 | 5 | 0.7762 | 0.9167±0.1286 | 0.2572 | 0.2123±0.0898 |
| 4 | chr3:2395000-2400000 | HE671395:17962-22962 | 5 | 0.7956 | 0.7747±0.1773 | 0.1704 | 0.1358±0.0374 |
| 5^b^ | chr3:6415000-6420000 | HE671062:78562-83562 | 5 | 0.8481 | 0.6127±0.2131 | 0.1240 | 0.1330±0.0515 |
| 6 | chr3:6965000-6970000 | HE670746:58196-63196 | 5 | 0.8694 | 0.6511±0.1305 | 0.2138 | 0.2235±0.0434 |
| 7 | chr4:700000-705000 | HE672065:10133-15133 | 5 | 0.7772 | 0.7299±0.1558 | 0.4375 | 0.4028±0.0887 |
| 8 | chr4:1895000-1900000 | HE669256:275358-280358 | 5 | 0.8587 | 0.5365±0.1389 | 0.1696 | 0.1911±0.0505 |
| 9 | chr4:5490000-5495000 | HE671448:623593-628593 | 5 | 0.8714 | 0.7542±0.1636 | 0.4472 | 0.3492±0.0668 |
| 10^b^ | chr5:6495000-6500000 | HE671659:43317-48317 | 5 | 0.9167 | 0.7170±0.2218 | 0.1243 | 0.2627±0.1240 |
| 11 | chr5:6580000-6585000 | HE669934:5702-10702 | 5 | 0.8255 | 0.4723±0.1599 | 0.1110 | 0.1219±0.0368 |
| 12 | chr6:1860000-1865000 | HE670850:55652-60652 | 5 | 0.9207 | 0.7438±0.1865 | 0.2779 | 0.2167±0.0559 |
| 13 | chr6:4390000-4395000 | HE671941:163959-168959 | 5 | 1.0000 | 1.0000±0.0000 | 0.3013 | 0.2598±0.0883 |
| 14^a,b^ | chr6:7655000-7660000 | HE671530:724-5724 | 5 | 0.7959 | 0.4424±0.2484 | 0.1274 | 0.1066±0.0400 |
| 15^a,b^ | chr6:8775000-8780000 | HE670182:27543-32543 | 5 | 0.7927 | 0.4976±0.2522 | 0.1702 | 0.1078±0.0476 |
| 16^a,b^ | chr6:10445000-10450000 | HE671438:246040-251040 | 5 | 0.9556 | 0.6047±0.2793 | 0.3716 | 0.1722±0.0806 |
| 17 | chr6:11110000-11115000 | HE671186:279186-284186 | 5 | 0.8347 | 0.7907±0.1558 | 0.0751 | 0.1407±0.0370 |
| 18 | chr6:12485000-12490000 | HE671449:40866-45866 | 5 | 0.8090 | 0.8472±0.1242 | 0.2120 | 0.2048±0.0531 |
| 19^b^ | chr7:220000-225000 | HE671631:9190-14190 | 5 | 1.0000 | 1.0000±0.0000 | 0.5957 | 0.2594±0.1189 |
| 20^a,b^ | chr7:3535000-3540000 | HE671059:75970-80970 | 5 | 0.8342 | 0.4345±0.3621 | 0.0720 | 0.0706±0.0400 |
| 21^b^ | chr7:7485000-7490000 | HE670796:115852-120852 | 5 | 0.8868 | 0.7082±0.1965 | 0.0846 | 0.0567±0.0224 |
| 22^b^ | chr8:1825000-1830000 | HE670161:29463-34463 | 5 | 0.7610 | 0.6745±0.2185 | 0.1956 | 0.1131±0.0472 |
| 23^a^ | chr8:2140000-2145000 | HE671690:104361-109361 | 5 | 0.8947 | 0.6650±0.2812 | 0.1826 | 0.1561±0.0497 |
| 24^a^ | chr8:6375000-6380000 | HE670742:518420-523420 | 5 | 0.9020 | 0.3271±0.2561 | 0.0990 | 0.1162±0.0407 |
| 25^b^ | chr9:2605000-2610000 | HE671317:79045-84045 | 5 | 0.8281 | 0.7192±0.1918 | 0.1699 | 0.1798±0.0800 |
| 26^a,b^ | chr10:150000-155000 | HE671375:150000-155000 | 5 | 0.9412 | 0.661±0.2653 | 0.1650 | 0.1355±0.0620 |
| 27 | chr10:300000-305000 | HE671375:300000-305000 | 5 | 1.0000 | 1.0000±0.0000 | 0.3087 | 0.2806±0.0811 |
| 28^a,b^ | chr10:6470000-6475000 | HE670336:32533-37533 | 5 | 0.9200 | 0.6710±0.2732 | 0.1140 | 0.0992±0.0441 |
| 29 | chr10:10395000-10400000 | HE672018:393827-398827 | 5 | 0.7634 | 0.5511±0.1927 | 0.1715 | 0.1475±0.0326 |
| 30^a^ | chr10:13740000-13745000 | HE671542:62579-67579 | 5 | 0.8142 | 0.3796±0.1524 | 0.1409 | 0.1053±0.0330 |
| 31^a^ | chr10:16685000-16690000 | HE670875:514247-519247 | 5 | 0.7451 | 0.2772±0.2271 | 0.0557 | 0.0598±0.0180 |
| 32^b^ | chr11:475000-480000 | HE671140:207921-212921 | 5 | 0.7552 | 0.4095±0.1418 | 0.0526 | 0.0712±0.0276 |
| 33^a^ | chr12:1285000-1290000 | HE670583:296316-301316 | 5 | 0.7414 | 0.5100±0.3409 | 0.3374 | 0.2702±0.0911 |
| 34 | chr12:6280000-6285000 | HE671289:156614-161614 | 5 | 0.8641 | 0.5293±0.1737 | 0.1710 | 0.1019±0.0332 |
| 35^a^ | chr12:14700000-14705000 | HE671996:114009-119009 | 5 | 0.7866 | 0.4306±0.4306 | 0.1164 | 0.1845±0.0589 |
| 36^a^ | chr12:15680000-15685000 | HE670557:265233-270233 | 5 | 0.8688 | 0.7036±0.2678 | 0.0944 | 0.0835±0.0308 |
| 37 | chr13:4785000-4790000 | HE671822:93367-98367 | 5 | 0.7680 | 0.8507±0.1604 | 0.1813 | 0.2239±0.0651 |
| 38^a,b^ | chr13:6290000-6295000 | HE671804:94485-99485 | 5 | 0.7949 | 0.4318±0.3596 | 0.1439 | 0.1652±0.0800 |
| 39^b^ | chr13:8285000-8290000 | HE671394:57001-62001 | 5 | 0.7582 | 0.4892±0.1505 | 0.3342 | 0.1857±0.0923 |
| 40^a,b^ | chr13:8335000-8340000 | HE671611:32515-37515 | 5 | 0.8571 | 0.4000±0.4690 | 0.0861 | 0.0611±0.0353 |
| 41 | chr13:11675000-11680000 | HE671818:16514-21514 | 5 | 0.7586 | 0.5987±0.1339 | 0.0995 | 0.1139±0.0415 |
| 42^a,b^ | chr14:6675000-6680000 | HE671569:22263-27263 | 5 | 0.7778 | 0.6637±0.2784 | 0.0652 | 0.0709±0.0287 |
| 43^a,b^ | chr15:2790000-2795000 | HE671917:71289-76289 | 5 | 0.8754 | 0.1822±0.4421 | 0.1174 | 0.0773±0.0541 |
| 44^a,b^ | chr16:2905000-2910000 | HE669883:31983-36983 | 5 | 0.7582 | 0.5077±0.2933 | 0.3800 | 0.3543±0.1852 |
| 45 | chr16:8895000-8900000 | HE667855:42347-47347 | 5 | 0.8464 | 0.6481±0.0980 | 0.2111 | 0.1747±0.0336 |
| 46^b^ | chr17:400000-405000 | HE671574:283482-288482 | 5 | 0.9143 | 0.7169±0.1629 | 0.2446 | 0.1746±0.0654 |
| 47^a,b^ | chr17:1695000-1700000 | HE671441:172147-177147 | 5 | 0.9512 | 0.6003±0.2852 | 0.1775 | 0.1544±0.0748 |
| 48^a^ | chr17:3810000-3815000 | HE670307:43194-48194 | 5 | 0.8667 | 0.2057±0.4432 | 0.1495 | 0.1261±0.0379 |
| 49^a^ | chr17:4795000-4800000 | HE671591:341237-346237 | 5 | 0.7959 | 0.1753±0.3074 | 0.0693 | 0.0786±0.0232 |
| 50 | chr17:5130000-5135000 | HE671591:676237-681237 | 5 | 0.7612 | 0.5677±0.1465 | 0.0696 | 0.0932±0.0222 |
| 51^a^ | chr17:12655000-12660000 | HE669583:66462-71462 | 5 | 0.9231 | 0.6133±0.2673 | 0.0552 | 0.0586±0.0213 |
| 52^b^ | chr18:400000-405000 | HE671969:95491-100491 | 5 | 0.9625 | 0.9744±0.0303 | 0.0494 | 0.0541±0.0239 |
| 53 | chr18:635000-640000 | HE671969:330491-335491 | 5 | 0.7548 | 0.6873±0.1873 | 0.0977 | 0.0871±0.0237 |
| 54 | chr18:5425000-5430000 | HE670289:23055-28055 | 5 | 0.8661 | 0.8330±0.1191 | 0.1005 | 0.1035±0.0347 |
| 55^a,b^ | chr18:15015000-15020000 | HE669517:56105-61105 | 5 | 0.7768 | 0.1757±0.3568 | 0.0334 | 0.0758±0.0413 |
| 56^a^ | chr18:15545000-15550000 | HE672049:59043-64043 | 5 | 0.7856 | 0.2446±0.2051 | 0.0944 | 0.0972±0.0294 |
| 57 | chr19:6870000-6875000 | HE672046:136-5136 | 5 | 0.7523 | 0.5887±0.2091 | 0.2261 | 0.2247±0.0471 |
| 58^a^ | chrZ:0-5000 | HE672038:1-5000 | 5 | 0.7605 | 0.3060±0.3525 | 0.0382 | 0.0548±0.0182 |
| 59^a^ | chrZ:3555000-3560000 | HE672037:12652-17652 | 5 | 0.7645 | 0.4455±0.3509 | 0.1186 | 0.1452±0.0443 |
| 60^a,b^ | chrZ:3990000-3995000 | HE671531:41749-46749 | 5 | 0.7692 | 0.6700±0.2705 | 0.0501 | 0.0943±0.0508 |
| 61 | chr1:8850000-8860000 | HE672073:240785-250785 | 10 | 0.6122 | 0.5057±0.1513 | 0.2518 | 0.2271±0.0400 |
| 62 | chr1:15010000-15020000 | HE671914:85876-95876 | 10 | 0.5800 | 0.4903±0.1288 | 0.1197 | 0.1182±0.0235 |
| 63 | chr2:150000-160000 | HE671404:40206-50206 | 10 | 0.5662 | 0.5243±0.1439 | 0.2847 | 0.2108±0.0384 |
| 64 ^a^ | chr2:440000-450000 | HE671428:17364-27364 | 10 | 0.6212 | 0.2940±0.1835 | 0.1214 | 0.1427±0.0336 |
| 65 | chr2:1880000-1890000 | HE672041:90176-100176 | 10 | 0.6762 | 0.5863±0.1726 | 0.2424 | 0.1629±0.0586 |
| 66 | chr2:1910000-1920000 | HE672041:120176-130176 | 10 | 0.5830 | 0.6774±0.1297 | 0.2514 | 0.1648±0.0332 |
| 67^a^ | chr6:10670000-10680000 | HE671859:136262-146262 | 10 | 0.6681 | 0.5755±0.2721 | 0.2042 | 0.1363±0.0439 |
| 68^a,b^ | chr7:7820000-7830000 | HE670389:81638-91638 | 10 | 0.8462 | 0.4349±0.2823 | 0.0873 | 0.0946±0.0415 |
| 69^a^ | chr7:11380000-11390000 | HE669356:8790-18790 | 10 | 0.6324 | 0.368±0.1649 | 0.1117 | 0.1065±0.0254 |
| 70^a^ | chr8:2700000-2710000 | HE670859:109395-119395 | 10 | 0.5772 | 0.4203±0.2388 | 0.1577 | 0.1128±0.0389 |
| 71 | chr10:11830000-11840000 | HE671341:728179-738179 | 10 | 0.5729 | 0.434±0.0738 | 0.2554 | 0.2496±0.0252 |
| 72 | chr12:840000-850000 | HE671156:118477-128477 | 10 | 0.6338 | 0.3464±0.0896 | 0.4011 | 0.3723±0.0452 |
| 73 | chr12:850000-860000 | HE671156:128477-138477 | 10 | 0.8058 | 0.5822±0.0904 | 0.4690 | 0.3729±0.0493 |
| 74 | chr12:5030000-5040000 | HE671858:12754-22754 | 10 | 0.6685 | 0.6480±0.2181 | 0.3722 | 0.3214±0.0833 |
| 75 | chr12:15840000-15850000 | HE670557:425233-435233 | 10 | 0.6485 | 0.4000±0.1250 | 0.1555 | 0.1337±0.0242 |
| 76 | chr13:280000-290000 | HE670462:42188-52188 | 10 | 0.5986 | 0.5807±0.1192 | 0.1819 | 0.1918±0.0293 |
| 77 | chr14:890000-900000 | HE671459:112697-122697 | 10 | 0.6072 | 0.4858±0.1081 | 0.2057 | 0.1699±0.0242 |
| 78 | chr15:7450000-7460000 | HE671383:51218-61218 | 10 | 0.7218 | 0.5818±0.0985 | 0.5774 | 0.4308±0.0487 |
| 79 | chr16:320000-330000 | HE671439:139401-149401 | 10 | 0.5721 | 0.5648±0.1185 | 0.2444 | 0.2209±0.0425 |
| 80 | chr16:9020000-9030000 | HE671226:97586-107586 | 10 | 0.6141 | 0.5625±0.1145 | 0.1920 | 0.1640±0.0333 |
| 81^a^ | chr16:9280000-9290000 | HE671647:79638-89638 | 10 | 0.5831 | 0.1514±0.2092 | 0.0352 | 0.0498±0.0159 |
| 82 | chr18:140000-150000 | HE671865:140000-150000 | 10 | 0.5920 | 0.4291±0.1210 | 0.1704 | 0.2115±0.0506 |
| 83 | chr18:3670000-3680000 | HE671488:971741-981741 | 10 | 0.6104 | 0.4931±0.1590 | 0.1241 | 0.1186±0.0223 |
| 84 | chr18:14400000-14410000 | HE671975:123370-133370 | 10 | 0.5955 | 0.3805±0.1148 | 0.2336 | 0.2234±0.0253 |
| 85 | chr18:14410000-14420000 | HE671975:133370-143370 | 10 | 0.6745 | 0.5794±0.1054 | 0.1813 | 0.1665±0.0293 |
| 86^a^ | chr19:30000-40000 | HE670819:30000-40000 | 10 | 0.5971 | 0.2803±0.2405 | 0.1821 | 0.1761±0.0512 |
| 87 | chr19:9790000-9800000 | HE669843:33987-43987 | 10 | 0.6067 | 0.6583±0.0923 | 0.1544 | 0.1396±0.0362 |
| 88 | chr19:11320000-11330000 | HE670722:35821-45821 | 10 | 0.6500 | 0.6017±0.1396 | 0.0713 | 0.0681±0.0231 |
| 89 | chr19:11770000-11780000 | HE670829:29944-39944 | 10 | 0.5737 | 0.5018±0.1835 | 0.0788 | 0.0903±0.0228 |
| 90^a^ | chr20:130000-140000 | HE670477:97268-107268 | 10 | 0.6901 | 0.4989±0.1886 | 0.3796 | 0.3314±0.0680 |
| 91 | chr20:3980000-3990000 | HE670907:120779-130779 | 10 | 0.5861 | 0.4966±0.1800 | 0.2610 | 0.2474±0.0408 |
| 92 | chrZ:210000-220000 | HE672038:210000-220000 | 10 | 0.5970 | 0.4013±0.1301 | 0.1262 | 0.1809±0.0377 |
| 93 | chr1:12150000-12200000 | HE667922:62931-112931 | 50 | 0.3897 | 0.6566±0.1340 | 0.1314 | 0.1254±0.0142 |
| 94 | chr11:9800000-9850000 | HE672060:343196-393196 | 50 | 0.4483 | 0.2952±0.0775 | 0.2358 | 0.2072±0.0186 |
| 95 | chr11:9850000-9900000 | HE672060:393196-443196 | 50 | 0.7113 | 0.4711±0.0663 | 0.4370 | 0.2953±0.0235 |
| 96 | chr18:1150000-1200000 | HE670865:305844-355844 | 50 | 0.4883 | 0.3521±0.0808 | 0.1553 | 0.1434±0.0207 |
| 97 | chr18:1200000-1250000 | HE670865:355844-405844 | 50 | 0.3468 | 0.2422±0.0667 | 0.1900 | 0.1747±0.0156 |

*D* denotes *D* (*H.* *numata*, *H.* *besckei*, *H.* *m. nanna*, *H.* *wallacei*)

*D* mean denotes the arithmetic mean of *D* with standard error of each given region

*f_d_* denotes *f_d_* (*H.* *numata*, *H.* *besckei*, *H.* *m. nanna*, *H.* *wallacei*)

*f_d_* mean denotes the arithmetic mean of *f_d_* with standard error of each given region

^a^ Locus filtered out because FDR adjusted *P*-value of mean *D* > 0.01

^b^ Locus filtered out because FDR adjustedP-value mean *f_d_* > 0.01

**Table S4.** Results of chromosomal d_xy_.

|  | **mean d_xy_**  **(*besckei, numata*)** | **mean d_xy_**  **(*m. nanna, numata*)** | **mean d_xy_**  **(*besckei, m. nanna*)** |
| --- | --- | --- | --- |
| chr1 | 0.0247±0.0003 | 0.0243±0.0003 | 0.0273±0.0002 |
| chr2 | 0.0219±0.0007 | 0.0212±0.0007 | 0.0236±0.0007 |
| chr3 | 0.0219±0.0004 | 0.0213±0.0004 | 0.0240±0.0004 |
| chr4 | 0.0234±0.0005 | 0.0225±0.0004 | 0.0254±0.0005 |
| chr5 | 0.0233±0.0005 | 0.0224±0.0004 | 0.0254±0.0005 |
| chr6 | 0.0227±0.0003 | 0.0219±0.0003 | 0.0248±0.0004 |
| chr7 | 0.0229±0.0004 | 0.0221±0.0003 | 0.0250±0.0003 |
| chr8 | 0.0215±0.0004 | 0.0210±0.0004 | 0.0235±0.0004 |
| chr9 | 0.0249±0.0004 | 0.0237±0.0004 | 0.0267±0.0004 |
| chr10 | 0.0249±0.0003 | 0.0240±0.0002 | 0.0271±0.0003 |
| chr11 | 0.0240±0.0004 | 0.0231±0.0003 | 0.0262±0.0004 |
| chr12 | 0.0237±0.0003 | 0.0234±0.0003 | 0.0263±0.0003 |
| chr13 | 0.0217±0.0003 | 0.0212±0.0003 | 0.0240±0.0003 |
| chr14 | 0.0216±0.0005 | 0.0209±0.0004 | 0.0233±0.0005 |
| chr15 | 0.0210±0.0004 | 0.0207±0.0004 | 0.0230±0.0005 |
| chr16 | 0.0236±0.0004 | 0.0223±0.0004 | 0.0252±0.0004 |
| chr17 | 0.0226±0.0003 | 0.0220±0.0003 | 0.0250±0.0003 |
| chr18 | 0.0244±0.0003 | 0.0236±0.0003 | 0.0267±0.0003 |
| chr19 | 0.0228±0.0003 | 0.0226±0.0003 | 0.0251±0.0003 |
| chr20 | 0.0232±0.0005 | 0.0225±0.0004 | 0.0254±0.0005 |
| chrZ | 0.0194±0.0006 | 0.0218±0.0006 | 0.0234±0.0006 |

**Table S5.** Results of d_xy_ for all the candidate introgression loci.

| **ID** | **Chr** | **d_xy_**  **(*besckei,* *numata*)** | **d_xy_**  **(*numata*, *m.* *nanna*)** | **d_xy_**  **(*besckei*, *m.* *nanna*)** |
| --- | --- | --- | --- | --- |
| 1 | chr1:6425000-6430000 | 0.0153±0.0017 (chr1:0.0247±0.0003) | 0.0153±0.0017 (chr1:0.0243±0.0003) | 0.0152±0.0025 (chr1:0.0273±0.0002) |
| 2 | chr1:15495000-15500000 | 0.0044±0.0024 (chr1:0.0247±0.0003) | 0.0053±0.0027 (chr1:0.0243±0.0003) | 0.0070±0.0034 (chr1:0.0273±0.0002) |
| 3 | chr2:120000-125000 | 0.0099±0.0046 (chr2:0.0219±0.0007) | 0.0104±0.0050 (chr2:0.0212±0.0007) | 0.0097±0.0042 (chr2:0.0236±0.0007) |
| 4 | chr3:2395000-2400000 | 0.0093±0.0034 (chr3:0.0219±0.0004) | 0.0087±0.0034 (chr3:0.0213±0.0004) | 0.0083±0.0030 (chr3:0.0240±0.0004) |
| 5 | chr3:6415000-6420000 | 0.0055±0.0034 (chr3:0.0219±0.0004) | 0.0052±0.0034 (chr3:0.0213±0.0004) | 0.0068±0.0047 (chr3:0.0240±0.0004) |
| 6 | chr3:6965000-6970000 | 0.0137±0.0043 (chr3:0.0219±0.0004) | 0.0112±0.0034 (chr3:0.0213±0.0004) | 0.0119±0.0032 (chr3:0.0240±0.0004) |
| 7 | chr4:700000-705000 | 0.0069±0.0034 (chr4:0.0234±0.0005) | 0.0070±0.0038 (chr4:0.0225±0.0004) | 0.0052±0.0020 (chr4:0.0254±0.0005) |
| 8 | chr4:1895000-1900000 | 0.0072±0.0032 (chr4:0.0234±0.0005) | 0.0064±0.0029 (chr4:0.0225±0.0004) | 0.0060±0.0031 (chr4:0.0254±0.0005) |
| 9 | chr4:5490000-5495000 | 0.0135±0.0063 (chr4:0.0234±0.0005) | 0.0185±0.0084 (chr4:0.0225±0.0004) | 0.0106±0.0048 (chr4:0.0254±0.0005) |
| 10 | chr5:6495000-6500000 | 0.0045±0.0025 (chr5:0.0233±0.0005) | 0.0064±0.0033 (chr5:0.0224±0.0004) | 0.0060±0.0032 (chr5:0.0254±0.0005) |
| 11 | chr5:6580000-6585000 | 0.0112±0.0045 (chr5:0.0233±0.0005) | 0.0124±0.0048 (chr5:0.0224±0.0004) | 0.0078±0.0032 (chr5:0.0254±0.0005) |
| 12 | chr6:1860000-1865000 | 0.0085±0.0022 (chr6:0.0227±0.0003) | 0.0098±0.0023 (chr6:0.0219±0.0003) | 0.0083±0.0021 (chr6:0.0248±0.0004) |
| 13 | chr6:4390000-4395000 | 0.0084±0.0038 (chr6:0.0227±0.0003) | 0.0099±0.0040 (chr6:0.0219±0.0003) | 0.0105±0.0041 (chr6:0.0248±0.0004) |
| 14 | chr6:7655000-7660000 | 0.0023±0.0015 (chr6:0.0227±0.0003) | 0.0027±0.0017 (chr6:0.0219±0.0003) | 0.0025±0.0017 (chr6:0.0248±0.0004) |
| 15 | chr6:8775000-8780000 | 0.0062±0.0026 (chr6:0.0227±0.0003) | 0.0060±0.0029 (chr6:0.0219±0.0003) | 0.0070±0.0028 (chr6:0.0248±0.0004) |
| 16 | chr6:10445000-10450000 | 0.0017±0.0008 (chr6:0.0227±0.0003) | 0.0014±0.0006 (chr6:0.0219±0.0003) | 0.0015±0.0006 (chr6:0.0248±0.0004) |
| 17 | chr6:11110000-11115000 | 0.0075±0.0025 (chr6:0.0227±0.0003) | 0.0106±0.0041 (chr6:0.0219±0.0003) | 0.0129±0.0044 (chr6:0.0248±0.0004) |
| 18 | chr6:12485000-12490000 | 0.0168±0.0034 (chr6:0.0227±0.0003) | 0.0198±0.0039 (chr6:0.0219±0.0003) | 0.0163±0.0029 (chr6:0.0248±0.0004) |
| 19 | chr7:220000-225000 | 0.0014±0.0009 (chr7:0.0229±0.0004) | 0.0011±0.0008 (chr7:0.0221±0.0003) | 0.0011±0.0007 (chr7:0.0250±0.0003) |
| 20 | chr7:3535000-3540000 | 0.0093±0.0028 (chr7:0.0229±0.0004) | 0.0099±0.0031 (chr7:0.0221±0.0003) | 0.0119±0.0034 (chr7:0.0250±0.0003) |
| 21 | chr7:7485000-7490000 | 0.0130±0.0048 (chr7:0.0229±0.0004) | 0.0097±0.0038 (chr7:0.0221±0.0003) | 0.0097±0.0038 (chr7:0.0250±0.0003) |
| 22 | chr8:1825000-1830000 | 0.0099±0.0048 (chr8:0.0215±0.0004) | 0.0136±0.0056 (chr8:0.0210±0.0004) | 0.0118±0.0048 (chr8:0.0235±0.0004) |
| 23 | chr8:2140000-2145000 | 0.0019±0.0011 (chr8:0.0215±0.0004) | 0.0025±0.0013 (chr8:0.0210±0.0004) | 0.0026±0.0014 (chr8:0.0235±0.0004) |
| 24 | chr8:6375000-6380000 | 0.0121±0.0024 (chr8:0.0215±0.0004) | 0.0119±0.0023 (chr8:0.0210±0.0004) | 0.0111±0.0025 (chr8:0.0235±0.0004) |
| 25 | chr9:2605000-2610000 | 0.0063±0.0031 (chr9:0.0207±0.0003) | 0.0060±0.0028 (chr9:0.0196±0.0003) | 0.0037±0.0018 (chr9:0.0235±0.0003) |
| 26 | chr10:150000-155000 | 0.0054±0.0018 (chr10:0.0249±0.0003) | 0.0059±0.0017 (chr10:0.0240±0.0002) | 0.0077±0.0021 (chr10:0.0271±0.0003) |
| 27 | chr10:300000-305000 | 0.0176±0.0066 (chr10:0.0249±0.0003) | 0.0187±0.0062 (chr10:0.0240±0.0002) | 0.0082±0.0026 (chr10:0.0271±0.0003) |
| 28 | chr10:6470000-6475000 | 0.0077±0.0038 (chr10:0.0249±0.0003) | 0.0093±0.0048 (chr10:0.0240±0.0002) | 0.0084±0.0039 (chr10:0.0271±0.0003) |
| 29^a^ | chr10:10395000-10400000 | 0.0240±0.0041 (chr10:0.0249±0.0003) | 0.0279±0.0033 (chr10:0.0240±0.0002) | 0.0228±0.0024 (chr10:0.0271±0.0003) |
| 30 | chr10:13740000-13745000 | 0.0153±0.0038 (chr10:0.0249±0.0003) | 0.0185±0.0048 (chr10:0.0240±0.0002) | 0.0189±0.0052 (chr10:0.0271±0.0003) |
| 31^a^ | chr10:16685000-16690000 | 0.0156±0.0028 (chr10:0.0249±0.0003) | 0.0308±0.0034 (chr10:0.0240±0.0002) | 0.0286±0.0032 (chr10:0.0271±0.0003) |
| 32 | chr11:475000-480000 | 0.0061±0.0032 (chr11:0.0240±0.0004) | 0.0065±0.0038 (chr11:0.0231±0.0003) | 0.0071±0.0044 (chr11:0.0262±0.0004) |
| 33 | chr12:1285000-1290000 | 0.0021±0.0021 (chr12:0.0237±0.0003) | 0.0023±0.0024 (chr12:0.0234±0.0003) | 0.0024±0.0026 (chr12:0.0263±0.0003) |
| 34 | chr12:6280000-6285000 | 0.0092±0.0039 (chr12:0.0237±0.0003) | 0.0083±0.0035 (chr12:0.0234±0.0003) | 0.0085±0.0035 (chr12:0.0263±0.0003) |
| 35^a^ | chr12:14700000-14705000 | 0.0205±0.0034 (chr12:0.0237±0.0003) | 0.0243±0.0043 (chr12:0.0234±0.0003) | 0.0245±0.0044 (chr12:0.0263±0.0003) |
| 36^a^ | chr12:15680000-15685000 | 0.0167±0.0046 (chr12:0.0237±0.0003) | 0.0175±0.0043 (chr12:0.0234±0.0003) | 0.0198±0.0051 (chr12:0.0263±0.0003) |
| 37 | chr13:4785000-4790000 | 0.0063±0.0028 (chr13:0.0217±0.0003) | 0.0056±0.0027 (chr13:0.0212±0.0003) | 0.0070±0.0032 (chr13:0.0240±0.0003) |
| 38 | chr13:6290000-6295000 | 0.0078±0.0043 (chr13:0.0217±0.0003) | 0.0069±0.0035 (chr13:0.0212±0.0003) | 0.0072±0.0039 (chr13:0.0240±0.0003) |
| 39 | chr13:8285000-8290000 | 0.0001±0.0001 (chr13:0.0217±0.0003) | 0.0001±0.0002 (chr13:0.0212±0.0003) | 0.0001±0.0002 (chr13:0.0240±0.0003) |
| 40 | chr13:8335000-8340000 | 0.0031±0.0015 (chr13:0.0217±0.0003) | 0.0069±0.0035 (chr13:0.0212±0.0003) | 0.0075±0.0036 (chr13:0.0240±0.0003) |
| 41 | chr13:11675000-11680000 | 0.0031±0.0015 (chr13:0.0217±0.0003) | 0.0071±0.0034 (chr13:0.0212±0.0003) | 0.0073±0.0036 (chr13:0.0240±0.0003) |
| 42 | chr14:6675000-6680000 | 0.0103±0.0028 (chr14:0.0216±0.0005) | 0.0082±0.0023 (chr14:0.0209±0.0004) | 0.0106±0.0031 (chr14:0.0233±0.0005) |
| 43 | chr15:2790000-2795000 | 0.0029±0.0016 (chr15:0.0210±0.0004) | 0.0029±0.0016 (chr15:0.0207±0.0004) | 0.0031±0.0017 (chr15:0.0230±0.0005) |
| 44 | chr16:2905000-2910000 | 0.0025±0.0030 (chr16:0.0236±0.0004) | 0.0026±0.0031 (chr16:0.0223±0.0004) | 0.0023±0.0022 (chr16:0.0252±0.0004) |
| 45 | chr16:8895000-8900000 | 0.0100±0.0040 (chr16:0.0236±0.0004) | 0.0112±0.0051 (chr16:0.0223±0.0004) | 0.0099±0.0038 (chr16:0.0252±0.0004) |
| 46 | chr17:400000-405000 | 0.0042±0.0026 (chr17:0.0226±0.0003) | 0.0040±0.0025 (chr17:0.0220±0.0003) | 0.0040±0.0025 (chr17:0.0250±0.0003) |
| 47 | chr17:1695000-1700000 | 0.0033±0.0024 (chr17:0.0226±0.0003) | 0.0029±0.0022 (chr17:0.0220±0.0003) | 0.0015±0.0012 (chr17:0.0250±0.0003) |
| 48 | chr17:3810000-3815000 | 0.0020±0.0014 (chr17:0.0226±0.0003) | 0.0013±0.0013 (chr17:0.0220±0.0003) | 0.0016±0.0014 (chr17:0.0250±0.0003) |
| 49 | chr17:4795000-4800000 | 0.0147±0.0025 (chr17:0.0226±0.0003) | 0.0164±0.0029 (chr17:0.0220±0.0003) | 0.0168±0.0031 (chr17:0.0250±0.0003) |
| 50^a^ | chr17:5130000-5135000 | 0.0191±0.0028 (chr17:0.0226±0.0003) | 0.0181±0.0027 (chr17:0.0220±0.0003) | 0.0204±0.0028 (chr17:0.0250±0.0003) |
| 51 | chr17:12655000-12660000 | 0.0097±0.0018 (chr17:0.0226±0.0003) | 0.0146±0.0031 (chr17:0.0220±0.0003) | 0.0134±0.0027 (chr17:0.0250±0.0003) |
| 52 | chr18:400000-405000 | 0.0103±0.0025 (chr18:0.0244±0.0003) | 0.0104±0.0028 (chr18:0.0236±0.0003) | 0.0106±0.0028 (chr18:0.0267±0.0003) |
| 53 | chr18:635000-640000 | 0.0074±0.0021 (chr18:0.0244±0.0003) | 0.0126±0.0032 (chr18:0.0236±0.0003) | 0.0136±0.0032 (chr18:0.0267±0.0003) |
| 54 | chr18:5425000-5430000 | 0.0119±0.0025 (chr18:0.0244±0.0003) | 0.0187±0.0044 (chr18:0.0236±0.0003) | 0.0210±0.0047 (chr18:0.0276±0.0003) |
| 55 | chr18:15015000-15020000 | 0.0102±0.0019 (chr18:0.0244±0.0003) | 0.0151±0.0031 (chr18:0.0236±0.0003) | 0.0168±0.0031 (chr18:0.0267±0.0003) |
| 56 | chr18:15545000-15550000 | 0.0156±0.0030 (chr18:0.0244±0.0003) | 0.0131±0.0019 (chr18:0.0236±0.0003) | 0.0165±0.0027 (chr18:0.0267±0.0003) |
| 57 | chr19:6870000-6875000 | 0.0053±0.0028 (chr19:0.0228±0.0003) | 0.0044±0.0022 (chr19:0.0226±0.0003) | 0.0028±0.0015 (chr19:0.0251±0.0003) |
| 58 | chrZ:0-5000 | 0.0160±0.0033  (chrZ:0.0194±0.0006) | 0.0169±0.0036  (chrZ:0.0218±0.0006) | 0.0183±0.0036  (chrZ:0.0234±0.0006) |
| 59 | chrZ:3555000-3560000 | 0.0112±0.0041  (chrZ:0.0194±0.0006) | 0.0144±0.0045  (chrZ:0.0218±0.0006) | 0.0152±0.0057  (chrZ:0.0234±0.0006) |
| 60 | chrZ:3990000-3995000 | 0.0046±0.0024 (chrZ:0.0194±0.0006) | 0.0069±0.0031  (chrZ:0.0218±0.0006) | 0.0081±0.0037 (chrZ:0.0234±0.0006) |
| 61 | chr1:8850000-8860000 | 0.0148±0.0026 (chr1:0.0247±0.0003) | 0.0150±0.0023 (chr1:0.0243±0.0003) | 0.0112±0.0024 (chr1:0.0273±0.0002) |
| 62 | chr1:15010000-15020000 | 0.0124±0.0018 (chr1:0.0247±0.0003) | 0.0185±0.0024 (chr1:0.0243±0.0003) | 0.0182±0.0029 (chr1:0.0273±0.0002) |
| 63 | chr2:150000-160000 | 0.0099±0.0025 (chr2:0.0219±0.0007) | 0.0098±0.0025 (chr2:0.0212±0.0007) | 0.0077±0.0020 (chr2:0.0236±0.0007) |
| 64 | chr2:440000-450000 | 0.0110±0.0027 (chr2:0.0219±0.0007) | 0.0130±0.0033 (chr2:0.0212±0.0007) | 0.0119±0.0029 (chr2:0.0236±0.0007) |
| 65 | chr2:1880000-1890000 | 0.0013±0.0007 (chr2:0.0219±0.0007) | 0.0013±0.0007 (chr2:0.0212±0.0007) | 0.0008±0.0004 (chr2:0.0236±0.0007) |
| 66 | chr2:1910000-1920000 | 0.0038±0.0013 (chr2:0.0219±0.0007) | 0.0043±0.0014 (chr2:0.0212±0.0007) | 0.0028±0.0010 (chr2:0.0236±0.0007) |
| 67 | chr6:10670000-10680000 | 0.0036±0.0018 (chr6:0.0227±0.0003) | 0.0032±0.0017 (chr6:0.0219±0.0003) | 0.0030±0.0016 (chr6:0.0248±0.0003) |
| 68 | chr7:7820000-7830000 | 0.0020±0.0014 (chr7:0.0229±0.0004) | 0.0031±0.0020 (chr7:0.0221±0.0003) | 0.0032±0.0020 (chr7:0.0250±0.0003) |
| 69 | chr7:11380000-11390000 | 0.0121±0.0033 (chr7:0.0229±0.0004) | 0.0108±0.0027 (chr7:0.0221±0.0003) | 0.0135±0.0034 (chr7:0.0250±0.0003) |
| 70 | chr8:2700000-2710000 | 0.0035±0.0016 (chr8:0.0215±0.0004) | 0.0038±0.0017 (chr8:0.0210±0.0004) | 0.0030±0.0013 (chr8:0.0235±0.0004) |
| 71 | chr10:11830000-11840000 | 0.0238±0.0028 (chr10:0.0249±0.0003) | 0.0229±0.0024 (chr10:0.0240±0.0002) | 0.0166±0.0018 (chr10:0.0271±0.0003) |
| 72 | chr12:840000-850000 | 0.0150±0.0027 (chr12:0.0237±0.0003) | 0.0149±0.0029 (chr12:0.0234±0.0003) | 0.0095±0.0018 (chr12:0.0263±0.0003) |
| 73 | chr12:850000-860000 | 0.0075±0.0021 (chr12:0.0237±0.0003) | 0.0079±0.0022 (chr12:0.0234±0.0003) | 0.0039±0.0009 (chr12:0.0263±0.0003) |
| 74 | chr12:5030000-5040000 | 0.0053±0.0030 (chr12:0.0237±0.0003) | 0.0050±0.0030 (chr12:0.0234±0.0003) | 0.0022±0.0009 (chr12:0.0263±0.0003) |
| 75 | chr12:15840000-15850000 | 0.0172±0.0023 (chr12:0.0237±0.0003) | 0.0178±0.0025 (chr12:0.0234±0.0003) | 0.0150±0.0022 (chr12:0.0263±0.0003) |
| 76 | chr13:280000-290000 | 0.0176±0.0025 (chr13:0.0217±0.0003) | 0.0164±0.0023 (chr13:0.0212±0.0003) | 0.0145±0.0022 (chr13:0.0240±0.0003) |
| 77 | chr14:890000-900000 | 0.0186±0.0031 (chr14:0.0216±0.0005) | 0.0201±0.0036 (chr14:0.0209±0.0004) | 0.0174±0.0034 (chr14:0.0233±0.0005) |
| 78 | chr15:7450000-7460000 | 0.0114±0.0034 (chr15:0.0210±0.0004) | 0.0122±0.0037 (chr15:0.0207±0.0004) | 0.0057±0.0018 (chr15:0.0230±0.0005) |
| 79 | chr16:320000-330000 | 0.0133±0.0031 (chr16:0.0236±0.0004) | 0.0122±0.0029 (chr16:0.0223±0.0004) | 0.0086±0.0021 (chr16:0.0252±0.0004) |
| 80 | chr16:9020000-9030000 | 0.0077±0.0021 (chr16:0.0236±0.0004) | 0.0069±0.0017 (chr16:0.0223±0.0004) | 0.0071±0.0018 (chr16:0.0252±0.0004) |
| 81 | chr16:9280000-9290000 | 0.0099±0.0021 (chr16:0.0236±0.0004) | 0.0160±0.0028 (chr16:0.0223±0.0004) | 0.0191±0.0034 (chr16:0.0252±0.0004) |
| 82 | chr18:140000-150000 | 0.0063±0.0016 (chr18:0.0244±0.0003) | 0.0059±0.0015 (chr18:0.0236±0.0003) | 0.0068±0.0017 (chr18:0.0267±0.0003) |
| 83^a^ | chr18:3670000-3680000 | 0.0190±0.0021 (chr18:0.0244±0.0003) | 0.0255±0.0029 (chr18:0.0236±0.0003) | 0.0248±0.0028 (chr18:0.0267±0.0003) |
| 84 | chr18:14400000-14410000 | 0.0201±0.0028 (chr18:0.0244±0.0003) | 0.0216±0.0029 (chr18:0.0236±0.0003) | 0.0175±0.0022 (chr18:0.0267±0.0003) |
| 85 | chr18:14410000-14420000 | 0.0141±0.0029 (chr18:0.0244±0.0003) | 0.0149±0.0029 (chr18:0.0236±0.0003) | 0.0147±0.0033 (chr18:0.0267±0.0003) |
| 86 | chr19:30000-40000 | 0.0045±0.0013 (chr19:0.0228±0.0003) | 0.0044±0.0013 (chr19:0.0226±0.0003) | 0.0053±0.0019 (chr19:0.0251±0.0003) |
| 87 | chr19:9790000-9800000 | 0.0124±0.0025 (chr19:0.0228±0.0003) | 0.0119±0.0025 (chr19:0.0226±0.0003) | 0.0121±0.0017 (chr19:0.0251±0.0003) |
| 88 | chr19:11320000-11330000 | 0.0036±0.0011 (chr19:0.0228±0.0003) | 0.0073±0.0020 (chr19:0.0226±0.0003) | 0.0066±0.0019 (chr19:0.0251±0.0003) |
| 89 | chr19:11770000-11780000 | 0.0112±0.0013 (chr19:0.0228±0.0003) | 0.0178±0.0023 (chr19:0.0226±0.0003) | 0.0189±0.0021 (chr19:0.0251±0.0003) |
| 90 | chr20:130000-140000 | 0.0121±0.0025 (chr20:0.0232±0.0005) | 0.0110±0.0022 (chr20:0.0225±0.0004) | 0.0053±0.0013 (chr20:0.0254±0.0005) |
| 91 | chr20:3980000-3990000 | 0.0061±0.0021 (chr20:0.0232±0.0005) | 0.0060±0.0018 (chr20:0.0225±0.0004) | 0.0056±0.0018 (chr20:0.0254±0.0005) |
| 92 | chrZ:210000-220000 | 0.0090±0.0021  (chrZ:0.0194±0.0006) | 0.0107±0.0025  (chrZ:0.0218±0.0006) | 0.0095±0.0022  (chrZ:0.0234±0.0006) |
| 93 | chr1:12150000-12200000 | 0.0184±0.0012 (chr1:0.0247±0.0003) | 0.0211±0.0014 (chr1:0.0243±0.0003) | 0.0197±0.0013 (chr1:0.0273±0.0002) |
| 94 | chr11:9800000-9850000 | 0.0154±0.0012 (chr11:0.0240±0.0004) | 0.0155±0.0011 (chr11:0.0231±0.0003) | 0.0125±0.0009 (chr11:0.0262±0.0004) |
| 95 | chr11:9850000-9900000 | 0.0151±0.0014 (chr11:0.0240±0.0004) | 0.0149±0.0013 (chr11:0.0231±0.0003) | 0.0094±0.0011 (chr11:0.0262±0.0004) |
| 96 | chr18:1150000-1200000 | 0.0131±0.0013 (chr18:0.0244±0.0003) | 0.0133±0.0013 (chr18:0.0236±0.0003) | 0.0112±0.0011 (chr18:0.0267±0.0003) |
| 97 | chr18:1200000-1250000 | 0.0188±0.0013 (chr18:0.0244±0.0003) | 0.0200±0.0014 (chr18:0.0236±0.0003) | 0.0172±0.0015 (chr18:0.0267±0.0003) |

^a^ Locus filtered out because of FDR adjusted *P*-value of d_xy_ (*H. besckei*, *H. m. nanna*) > 0.01

**Table S6.** Mean sequence read depths across candidate introgression loci for the four focal species.

| **ID** | **Chr** | **Scaf** | **length (kb)** | **Read Depth** | | | | |
| --- | --- | --- | --- | --- | --- | --- | --- | --- |
|  |  |  |  | ***besckei*** | ***numata*** | ***m. nanna*** | | ***wallacei*** |
| 1 | chr1:6425000-6430000 | HE669357:205937-210937 | 5 | 14.5793 | 15.5883 | | 17.0516 | 26.3789 |
| 2 | chr1:15495000-15500000 | HE671415:36614-41614 | 5 | 14.5447 | 14.4525 | | 28.8843 | 6.02786 |
| 3 | chr2:120000-125000 | HE671404:10206-15206 | 5 | 9.05982 | 11.5544 | | 9.69085 | 5.39119 |
| 4 | chr3:2395000-2400000 | HE671395:17962-22962 | 5 | 11.7088 | 10.5869 | | 9.96265 | 10.3483 |
| 5^a^ | chr3:6415000-6420000 | HE671062:78562-83562 | 5 | 41.0598 | 17.6959 | | 42.2634 | 8.09821 |
| 6 | chr3:6965000-6970000 | HE670746:58196-63196 | 5 | 13.0453 | 11.697 | | 14.2477 | 14.9255 |
| 7 | chr4:700000-705000 | HE672065:10133-15133 | 5 | 8.16116 | 13.5065 | | 10.309 | 10.5209 |
| 8 | chr4:1895000-1900000 | HE669256:275358-280358 | 5 | 9.59154 | 12.5527 | | 15.1185 | 16.272 |
| 9^a^ | chr4:5490000-5495000 | HE671448:623593-628593 | 5 | 14.2282 | 7.87043 | | 81.8764 | 4.37562 |
| 10^a^ | chr5:6495000-6500000 | HE671659:43317-48317 | 5 | 26.0486 | 43.3697 | | 17.3747 | 13.7311 |
| 11 | chr5:6580000-6585000 | HE669934:5702-10702 | 5 | 11.7243 | 10.3527 | | 7.89845 | 10.4387 |
| 12^a^ | chr6:1860000-1865000 | HE670850:55652-60652 | 5 | 14.1234 | 14.7109 | | 15.649 | 2.80435 |
| 13^a^ | chr6:4390000-4395000 | HE671941:163959-168959 | 5 | 45.7924 | 6.63913 | | 33.2593 | 4.69891 |
| 14 | chr6:7655000-7660000 | HE671530:724-5724 | 5 | 11.4281 | 10.8153 | | 7.00286 | 9.47892 |
| 15 | chr6:8775000-8780000 | HE670182:27543-32543 | 5 | 12.3292 | 12.9008 | | 10.8176 | 18.2442 |
| 16^a^ | chr6:10445000-10450000 | HE671438:246040-251040 | 5 | 99.2867 | 48.9497 | | 23.5373 | 100.751 |
| 17 | chr6:11110000-11115000 | HE671186:279186-284186 | 5 | 11.6726 | 12.3915 | | 11.1423 | 5.77778 |
| 18 | chr6:12485000-12490000 | HE671449:40866-45866 | 5 | 15.4282 | 15.1844 | | 17.5986 | 8.3883 |
| 19^a^ | chr7:220000-225000 | HE671631:9190-14190 | 5 | 97.9656 | 19.4548 | | 161.555 | 3.05812 |
| 20 | chr7:3535000-3540000 | HE671059:75970-80970 | 5 | 11.1294 | 13.3623 | | 15.8254 | 9.15821 |
| 21 | chr7:7485000-7490000 | HE670796:115852-120852 | 5 | 13.4928 | 13.8887 | | 16.648 | 11.4286 |
| 22 | chr8:1825000-1830000 | HE670161:29463-34463 | 5 | 12.1438 | 7.70689 | | 14.8319 | 6.57115 |
| 23 | chr8:2140000-2145000 | HE671690:104361-109361 | 5 | 10.1232 | 10.5058 | | 10.3202 | 4.96837 |
| 24 | chr8:6375000-6380000 | HE670742:518420-523420 | 5 | 14.6922 | 15.0012 | | 15.9769 | 7.90959 |
| 25 | chr9:2605000-2610000 | HE671317:79045-84045 | 5 | 13.7466 | 8.51334 | | 8.25837 | 9.01045 |
| 26 | chr10:150000-155000 | HE671375:150000-155000 | 5 | 10.7531 | 12.5331 | | 11.8841 | 7.08708 |
| 27 | chr10:300000-305000 | HE671375:300000-305000 | 5 | 9.04707 | 14.8746 | | 9.77496 | 6.39119 |
| 28 | chr10:6470000-6475000 | HE670336:32533-37533 | 5 | 11.8217 | 14.9504 | | 11.724 | 8.63038 |
| 29 | chr10:10395000-10400000 | HE672018:393827-398827 | 5 | 15.3493 | 16.5495 | | 16.7148 | 17.1014 |
| 30 | chr10:13740000-13745000 | HE671542:62579-67579 | 5 | 12.0075 | 13.0132 | | 17.2974 | 7.30145 |
| 31 | chr10:16685000-16690000 | HE670875:514247-519247 | 5 | 15.6998 | 16.1792 | | 18.3163 | 32.2987 |
| 32 | chr11:475000-480000 | HE671140:207921-212921 | 5 | 12.0473 | 16.0901 | | 17.098 | 15.3619 |
| 33 | chr12:1285000-1290000 | HE670583:296316-301316 | 5 | 6.52183 | 9.86852 | | 8.00528 | 7.24472 |
| 34 | chr12:6280000-6285000 | HE671289:156614-161614 | 5 | 18.369 | 12.0342 | | 19.7936 | 10.6505 |
| 35 | chr12:14700000-14705000 | HE671996:114009-119009 | 5 | 13.8523 | 15.3602 | | 14.8148 | 9.93352 |
| 36 | chr12:15680000-15685000 | HE670557:265233-270233 | 5 | 9.73892 | 9.35644 | | 14.2289 | 10.4208 |
| 37 | chr13:4785000-4790000 | HE671822:93367-98367 | 5 | 7.65623 | 10.0984 | | 9.97796 | 6.14949 |
| 38 | chr13:6290000-6295000 | HE671804:94485-99485 | 5 | 11.1134 | 15.246 | | 13.7446 | 11.7748 |
| 39^a^ | chr13:8285000-8290000 | HE671394:57001-62001 | 5 | 42.4915 | 29.1179 | | 17.6233 | 17.8098 |
| 40 | chr13:8335000-8340000 | HE671611:32515-37515 | 5 | 8.46456 | 8.92505 | | 14.9894 | 15.0056 |
| 41 | chr13:11675000-11680000 | HE671818:16514-21514 | 5 | 5.70692 | 6.55214 | | 12.5841 | 8.84041 |
| 42 | chr14:6675000-6680000 | HE671569:22263-27263 | 5 | 14.5282 | 15.9649 | | 16.0959 | 15.7695 |
| 43 | chr15:2790000-2795000 | HE671917:71289-76289 | 5 | 11.5174 | 9.27216 | | 7.94742 | 8.33102 |
| 44 | chr16:2905000-2910000 | HE669883:31983-36983 | 5 | 13.4163 | 14.06 | | 16.6775 | 13.4226 |
| 45^a^ | chr16:8895000-8900000 | HE667855:42347-47347 | 5 | 61.057 | 23.6019 | | 25.942 | 44.9914 |
| 46 | chr17:400000-405000 | HE671574:283482-288482 | 5 | 13.2564 | 14.9309 | | 16.4512 | 7.48387 |
| 47 | chr17:1695000-1700000 | HE671441:172147-177147 | 5 | 23.4187 | 14.3297 | | 16.0128 | 11.3854 |
| 48 | chr17:3810000-3815000 | HE670307:43194-48194 | 5 | 11.1914 | 11.4596 | | 19.0684 | 5.28601 |
| 49 | chr17:4795000-4800000 | HE671591:341237-346237 | 5 | 11.9295 | 10.3346 | | 14.3339 | 9.41165 |
| 50 | chr17:5130000-5135000 | HE671591:676237-681237 | 5 | 14.8541 | 15.0781 | | 17.2002 | 16.2687 |
| 51 | chr17:12655000-12660000 | HE669583:66462-71462 | 5 | 14.9997 | 16.6437 | | 18.889 | 27.1115 |
| 52 | chr18:400000-405000 | HE671969:95491-100491 | 5 | 14.2578 | 20.0188 | | 21.9722 | 8.17638 |
| 53 | chr18:635000-640000 | HE671969:330491-335491 | 5 | 9.84144 | 10.7103 | | 17.8368 | 20.2464 |
| 54 | chr18:5425000-5430000 | HE670289:23055-28055 | 5 | 14.1351 | 14.4195 | | 12.6533 | 10.541 |
| 55 | chr18:15015000-15020000 | HE669517:56105-61105 | 5 | 16.1053 | 15.2533 | | 14.28 | 5.49421 |
| 56 | chr18:15545000-15550000 | HE672049:59043-64043 | 5 | 15.3904 | 15.9087 | | 17.0453 | 21.0063 |
| 57 | chr19:6870000-6875000 | HE672046:136-5136 | 5 | 10.2236 | 12.7975 | | 11.8482 | 13.8327 |
| 58 | chrZ:0-5000 | HE672038:1-5000 | 5 | 10.5617 | 10.6371 | | 14.7725 | 12.3542 |
| 59 | chrZ:3555000-3560000 | HE672037:12652-17652 | 5 | 12.4999 | 15.8070 | | 14.2313 | 13.8938 |
| 60 | chrZ:3990000-3995000 | HE671531:41749-46749 | 5 | 16.4774 | 20.1915 | | 16.0189 | 10.5993 |
| 61 | chr1:8850000-8860000 | HE672073:240785-250785 | 10 | 14.6571 | 15.3067 | | 13.9703 | 16.8895 |
| 62 | chr1:15010000-15020000 | HE671914:85876-95876 | 10 | 14.0587 | 13.9737 | | 17.0579 | 11.5608 |
| 63 | chr2:150000-160000 | HE671404:40206-50206 | 10 | 21.1625 | 15.3785 | | 21.1057 | 13.4057 |
| 64 | chr2:440000-450000 | HE671428:17364-27364 | 10 | 16.888 | 16.2294 | | 17.3441 | 24.7645 |
| 65 | chr2:1880000-1890000 | HE672041:90176-100176 | 10 | 17.8652 | 18.7923 | | 18.4913 | 13.2124 |
| 66^a^ | chr2:1910000-1920000 | HE672041:120176-130176 | 10 | 24.1442 | 16.0746 | | 41.4766 | 20.1322 |
| 67 | chr6:10670000-10680000 | HE671859:136262-146262 | 10 | 8.76376 | 10.5655 | | 8.89787 | 8.43024 |
| 68 | chr7:7820000-7830000 | HE670389:81638-91638 | 10 | 17.6263 | 35.0038 | | 26.3379 | 12.5946 |
| 69 | chr7:11380000-11390000 | HE669356:8790-18790 | 10 | 14.7002 | 14.6137 | | 15.3511 | 9.33394 |
| 70 | chr8:2700000-2710000 | HE670859:109395-119395 | 10 | 10.6655 | 11.6016 | | 9.80814 | 5.91635 |
| 71 | chr10:11830000-11840000 | HE671341:728179-738179 | 10 | 16.7961 | 15.665 | | 16.3468 | 15.7251 |
| 72 | chr12:840000-850000 | HE671156:118477-128477 | 10 | 14.6369 | 14.5719 | | 13.8828 | 11.7134 |
| 73 | chr12:850000-860000 | HE671156:128477-138477 | 10 | 11.56 | 13.5439 | | 12.1295 | 17.8798 |
| 74 | chr12:5030000-5040000 | HE671858:12754-22754 | 10 | 12.2031 | 13.0301 | | 19.7796 | 37.775 |
| 75 | chr12:15840000-15850000 | HE670557:425233-435233 | 10 | 13.911 | 16.1935 | | 15.0362 | 11.1225 |
| 76 | chr13:280000-290000 | HE670462:42188-52188 | 10 | 15.5367 | 16.1572 | | 15.2772 | 17.3725 |
| 77 | chr14:890000-900000 | HE671459:112697-122697 | 10 | 13.6245 | 13.211 | | 14.9267 | 9.85226 |
| 78^a^ | chr15:7450000-7460000 | HE671383:51218-61218 | 10 | 87.8896 | 15.6708 | | 13.6006 | 9.14905 |
| 79 | chr16:320000-330000 | HE671439:139401-149401 | 10 | 14.7959 | 14.7477 | | 16.2567 | 15.7494 |
| 80 | chr16:9020000-9030000 | HE671226:97586-107586 | 10 | 15.2829 | 18.6876 | | 17.9502 | 12.3663 |
| 81 | chr16:9280000-9290000 | HE671647:79638-89638 | 10 | 9.29964 | 9.76918 | | 16.5058 | 12.01 |
| 82 | chr18:140000-150000 | HE671865:140000-150000 | 10 | 15.5219 | 16.1619 | | 12.2201 | 9.7487 |
| 83 | chr18:3670000-3680000 | HE671488:971741-981741 | 10 | 15.3385 | 15.6315 | | 15.5888 | 18.0635 |
| 84 | chr18:14400000-14410000 | HE671975:123370-133370 | 10 | 16.6011 | 18.3819 | | 16.6659 | 12.789 |
| 85 | chr18:14410000-14420000 | HE671975:133370-143370 | 10 | 15.9068 | 16.14 | | 17.6358 | 13.8196 |
| 86 | chr19:30000-40000 | HE670819:30000-40000 | 10 | 24.6196 | 17.9251 | | 22.1908 | 12.4948 |
| 87 | chr19:9790000-9800000 | HE669843:33987-43987 | 10 | 15.3678 | 14.6703 | | 20.4574 | 12.8221 |
| 88 | chr19:11320000-11330000 | HE670722:35821-45821 | 10 | 13.4683 | 14.1272 | | 16.9395 | 14.2162 |
| 89 | chr19:11770000-11780000 | HE670829:29944-39944 | 10 | 17.4273 | 15.6378 | | 17.7584 | 19.6588 |
| 90^a^ | chr20:130000-140000 | HE670477:97268-107268 | 10 | 25.159 | 52.9067 | | 184.286 | 12.0029 |
| 91 | chr20:3980000-3990000 | HE670907:120779-130779 | 10 | 12.7526 | 14.2774 | | 9.05226 | 12.817 |
| 92 | chrZ:210000-220000 | HE672038:210000-220000 | 10 | 11.1526 | 10.8835 | | 17.0157 | 33.9667 |
| 93 | chr1:12150000-12200000 | HE667922:62931-112931 | 50 | 16.5775 | 16.0014 | | 18.54 | 17.6451 |
| 94 | chr11:9800000-9850000 | HE672060:343196-393196 | 50 | 16.5066 | 15.94 | | 16.6089 | 16.9826 |
| 95 | chr11:9850000-9900000 | HE672060:393196-443196 | 50 | 17.1466 | 15.254 | | 17.4497 | 17.2615 |
| 96 | chr18:1150000-1200000 | HE670865:305844-355844 | 50 | 15.9524 | 15.7081 | | 17.2273 | 12.0787 |
| 97 | chr18:1200000-1250000 | HE670865:355844-405844 | 50 | 14.1993 | 14.471 | | 16.6435 | 11.131 |
| ^a^ Locus filtered out because of read depth below 5 or above 40. | | | |  |  | |  |  |

**Table S7.** Results of *D*-statistics to examine gene flow between other species and *H. m. nanna.*

| **ID** | **Chr** | **Scaf** | **Length (kb)** | ***D*** | ***D* mean** |
| --- | --- | --- | --- | --- | --- |
|  |  |  |  | ***D* (*besckei*, *ethilla*, *m. nanna*, *wallacei*)** | |
| 7 | chr4:700000-705000 | HE672065:10133-15133 | 5 | 0.1806 | 0.2918±0.2887 |
| 17 | chr6:11110000-11115000 | HE671186:279186-284186 | 5 | -0.7421 | -0.6453±0.1558*** |
| 27 | chr10:300000-305000 | HE671375:300000-305000 | 5 | 0.6250 | 0.3250±0.4415 |
| 61 | chr1:8850000-8860000 | HE672073:240785-250785 | 10 | 0.8252 | 0.5748±0.1097*** |
| 63 | chr2:150000-160000 | HE671404:40206-50206 | 10 | 0.2306 | 0.1835±0.1686 |
| 65 | chr2:1880000-1890000 | HE672041:90176-100176 | 10 | 0.4457 | 0.2483±0.1553 |
| 71 | chr10:11830000-11840000 | HE671341:728179-738179 | 10 | 0.2083 | 0.1221±0.1167 |
| 72 | chr12:840000-850000 | HE671156:118477-128477 | 10 | 0.4516 | -0.0035±0.0755 |
| 73 | chr12:850000-860000 | HE671156:128477-138477 | 10 | 0.5668 | 0.3258±0.1156** |
| 75 | chr12:15840000-15850000 | HE670557:425233-435233 | 10 | 0.7775 | 0.3508±0.0879*** |
| 76 | chr13:280000-290000 | HE670462:42188-52188 | 10 | 0.3226 | -0.0132±0.1306 |
| 77 | chr14:890000-900000 | HE671459:112697-122697 | 10 | -0.2884 | -0.2833±0.1335* |
| 79 | chr16:320000-330000 | HE671439:139401-149401 | 10 | 0.5404 | 0.1458±0.1585 |
| 82 | chr18:140000-150000 | HE671865:140000-150000 | 10 | -0.7848 | -0.4253±0.1250*** |
| 85 | chr18:14410000-14420000 | HE671975:133370-143370 | 10 | -0.6092 | -0.5213±0.1132*** |
| 93 | chr1:12150000-12160000 | HE667922:62937-72931 | 10 | 0.1403 | -0.0135±0.1624 |
| 94 | chr11:9800000-9850000 | HE672060:343196-393196 | 50 | 0.7339 | 0.4372±0.0635*** |
| 95 | chr11:9850000-9900000 | HE672060:393196-443196 | 50 | -0.1880 | -0.0550±0.0593 |
| 96 | chr18:1160000-1170000 | HE670865:315844-325844 | 10 | -0.5701 | -0.4646±0.1194*** |
| 97 | chr18:1240000-1250000 | HE670865:395844-405844 | 10 | -0.8340 | -0.6924±0.0738*** |
|  |  |  |  | ***D* (*besckei*, *pardalinus*, *m. nanna*, *wallacei*)** | |
| 7 | chr4:700000-705000 | HE672065:10133-15133 | 5 | 0.6726 | 0.6794±0.2055*** |
| 17 | chr6:11110000-11115000 | HE671186:279186-284186 | 5 | -0.5506 | -0.2723±0.1051** |
| 27 | chr10:300000-305000 | HE671375:300000-305000 | 5 | 0.9636 | 0.8560±0.1748*** |
| 65 | chr2:1880000-1890000 | HE672041:90176-100176 | 10 | 0.1800 | 0.1546±0.2223 |
| 71 | chr10:11830000-11840000 | HE671341:728179-738179 | 10 | 0.3150 | 0.2646±0.1060* |
| 76 | chr13:280000-290000 | HE670462:42188-52188 | 10 | 0.5132 | 0.2827±0.1276* |
| 77 | chr14:890000-900000 | HE671459:112697-122697 | 10 | -0.4210 | -0.3949±0.0979*** |
| 79 | chr16:320000-330000 | HE671439:139401-149401 | 10 | 0.6665 | 0.4422±0.1443** |
| 80 | chr16:9020000-9030000 | HE671226:97586-107586 | 10 | 0.5689 | 0.4208±0.1913* |
| 84 | chr18:14400000-14410000 | HE671975:123370-133370 | 10 | -0.3850 | -0.3487±0.1109** |
| 85 | chr18:14410000-14420000 | HE671975:133370-143370 | 10 | -0.4061 | -0.2325±0.1298 |
| 91 | chr20:3980000-3990000 | HE670907:120779-130779 | 10 | 0.5842 | 0.4452±0.2048* |
| 93 | chr1:12150000-12160000 | HE667922:62937-72931 | 10 | 0.0408 | -0.1653±0.1342 |
| 94 | chr11:9800000-9850000 | HE672060:343196-393196 | 50 | 0.5628 | 0.3804±0.0645*** |
| 95 | chr11:9850000-9900000 | HE672060:393196-443196 | 50 | 0.1183 | 0.0705±0.0716 |
| 96 | chr18:1160000-1170000 | HE670865:315844-325844 | 10 | -0.5640 | -0.4451±0.1357** |
| 97 | chr18:1240000-1250000 | HE670865:395844-405844 | 10 | -0.8273 | -0.6954±0.0695*** |
|  |  |  |  | ***D* (*besckei*, *ismenius*, *m. nanna*, *wallacei*)** | |
| 17 | chr6:11110000-11115000 | HE671186:279186-284186 | 5 | 0.1097 | -0.2563±0.4501 |
| 77 | chr14:890000-900000 | HE671459:112697-122697 | 10 | -0.5907 | -0.4475±0.0980*** |
| 79 | chr16:320000-330000 | HE671439:139401-149401 | 10 | -0.6192 | -0.3921±0.1516** |
| 85 | chr18:14410000-14420000 | HE671975:133370-143370 | 10 | -0.6538 | -0.4972±0.1235*** |
| 91 | chr20:3980000-3990000 | HE670907:120779-130779 | 10 | -0.3127 | -0.2753±0.1575 |
| 93 | chr1:12150000-12160000 | HE667922:62937-72931 | 10 | -0.4519 | -0.3512±0.1816 |
| 96 | chr18:1160000-1170000 | HE670865:315844-325844 | 10 | -0.6290 | -0.5016±0.1187*** |
| 97 | chr18:1240000-1250000 | HE670865:395844-405844 | 10 | -0.8531 | -0.6695±0.0784*** |
|  |  |  |  | ***D* (*besckei*, *hecale*, *m. nanna*, *wallacei*)** | |
| 77 | chr14:890000-900000 | HE671459:112697-122697 | 10 | -0.2646 | -0.2050±0.1235 |
| 79 | chr16:320000-330000 | HE671439:139401-149401 | 10 | 0.4926 | 0.3221±0.1662 |
| 80 | chr16:9020000-9030000 | HE671226:97586-107586 | 10 | 0.6776 | 0.5171±0.1549*** |
| 85 | chr18:14410000-14420000 | HE671975:133370-143370 | 10 | -0.5264 | -0.5476±0.1203*** |
| 93 | chr1:12150000-12160000 | HE667922:62937-72931 | 10 | -0.0063 | -0.0017±0.2112 |
| 96 | chr18:1160000-1170000 | HE670865:315844-325844 | 10 | -0.5889 | -0.4599±0.1303*** |
| 97 | chr18:1240000-1250000 | HE670865:395844-405844 | 10 | -0.7874 | -0.6470±0.0728*** |
|  |  |  |  | ***D* (*pardalinus*, *hecale*, *m. nanna*, *wallacei*)** | |
| 71 | chr10:11830000-11840000 | HE671341:728179-738179 | 10 | 0.2796 | 0.2462±0.1193* |
| 95 | chr11:9850000-9900000 | HE672060:393196-443196 | 50 | 0.0731 | 0.0725±0.0623 |
|  |  |  |  | ***D* (*ethilla*, *hecale*, *m.* *nanna*, *wallacei*)** | |
| 65 | chr2:1880000-1890000 | HE672041:90176-100176 | 5 | -0.5978 | -0.2833±0.1782 |
|  |  |  |  | ***D* (*cydno* *alithea*, *m. nanna*, *besckei*, *wallacei*)** | |
| 82 | chr18:140000-150000 | HE671865:140000-150000 | 10 | -0.1322 | 0.3983±0.1973* |

* indicates p<0.05, ** indicates p<0.01,*** indicates p<0.001

*D* mean denotes the arithmetic mean of *D* with standard error of each given region

**Table S8.** Gene annotations of 41 candidate introgression loci.

| **ID** | **Location** | **Gene** | **Annotation** |
| --- | --- | --- | --- |
| 1 | chr1 | HMEL003956 | hypothetical protein KGM_19707 [Danaus plexippus] |
| 4 | chr3 | HMEL013807 | cuticlin-1 |
| 6 | chr3 | HMEL008211 | hypothetical protein TCDM_05191 [Trypanosoma cruzi Dm28c] |
| 7 | chr4 | HMEL017418 | sorbitol dehydrogenase |
| 8 | chr4 |  |  |
| 11 | chr5 | HMEL005194 | [centrosomal protein of 162 kDa](http://blast.ncbi.nlm.nih.gov/Blast.cgi#alnHdr_827559139) |
| 17 | chr6 | HMEL022597 | serine protease snake-like |
| 18 | chr6 | HMEL022577 | transmembrane protein 26-like |
| 27 | chr10 |  |  |
| 34 | chr12 |  |  |
| 37 | chr13 | HMEL015651 | Z band alternatively spliced PDZ-motif protein 66 |
| 41 | chr13 | HMEL017853 | bestrophin-3-like |
| 53 | chr18 |  |  |
| 54 | chr18 |  |  |
| 57 | chr19 | HMEL003752 | ATP-dependent RNA helicase |
| 61 | chr1 | HMEL012399 | lactase-phlorizin hydrolase |
| 62 | chr1 | HMEL006992 | chromobox-like protein 5 |
|  |  | HMEL006993 | dedicator of cytokinesis protein 7-like |
| 63 | chr2 | HMEL013949 | 3-oxoacyl-[acyl-carrier-protein] reductase 1 |
| 65 | chr2 | HMEL015513 | fatty acid synthase-like |
| 71 | chr10 | HMEL013353 | splicing factor 45 |
|  |  | HMEL013354 | serine/threonine-protein kinase greatwall-like |
| 72 | chr12 | HMEL011002 | sugar transporter 4 |
| 73 | chr12 |  |  |
| 74 | chr12 | HMEL010727 | tigger transposable element-derived protein 6-like |
|  |  | HMEL010728 | tigger transposable element-derived protein 6-like |
| 75 | chr12 | HMEL007296 | muscle RAS oncogene-like protein |
| 76 | chr13 | HMEL006600 | connector enhancer of kinase suppressor of ras 2 |
| 77 | chr14 | HMEL014590 | WD repeat-containing protein CG11141-like |
| 79 | chr16 | HMEL014296 | V-type proton ATPase catalytic subunit A |
|  |  | HMEL014297 | speckle targeted PIP5K1A-regulated poly(A) polymerase-like |
| 80 | chr16 | HMEL011800 | maelstrom |
|  |  | HMEL011801 | TPA: alpha1,6 fucosyltransferase |
| 82 | chr18 | HMEL009688 | [zinc finger C4H2 domain-containing protein [Bombyx mori]](http://blast.ncbi.nlm.nih.gov/Blast.cgi#alnHdr_827557162) |
| 84 | chr18 | HMEL013926 | inositol 1,4,5-trisphosphate receptor-like |
| 85 | chr18 | HMEL013926 | inositol 1,4,5-trisphosphate receptor-like |
| 87 | chr19 | HMEL004979 | transmembrane protein 145-like |
|  |  | HMEL004980 | phosphatidylinositol 3-kinase 60 |
| 88 | chr19 |  |  |
| 89 | chr19 | HMEL008748 | synaptojanin |
| 91 | chr20 | HMEL009444 | chromodomain helicase-DNA-binding protein Mi-2-like protein |
|  |  | HMEL009446 | protein downstream neighbor of son homolog |
| 92 | chrZ | HMEL011037 | hypothetical protein KGM_21587 [Danaus plexippus] |
| 93 | chr1 | HMEL002288 | collagen alpha-2IV chain protein |
|  |  | HMEL002289 | thioredoxin family Trp26 |
|  |  | HMEL002290 | selenoprotein T |
|  |  | HMEL002291 | WD repeat-containing protein 35-like |
|  |  | HMEL002293 | voltage-gated ion channel |
| 94 | chr11 | HMEL015415 | high affinity copper transporter |
|  |  | HMEL015416 | folylpolyglutamate synthase, mitochondrial |
|  |  | HMEL015417 | RNA polymerase II subunit B1 CTD phosphatase RPAP2-like |
|  |  | HMEL015418 | adenylate kinase 8-like |
|  |  | HMEL015419 | heat shock protein cognate 3 |
|  |  | HMEL015420 | nad-specific glutamate dehydrogenase |
|  |  | HMEL015421 | NAD-specific glutamate dehydrogenase |
|  |  | HMEL015422 | NAD-specific glutamate dehydrogenase |
|  |  | HMEL015423 | heat shock cognate 70 protein |
|  |  | HMEL015424 | histone-lysine N-methyltransferase EHMT1-like |
|  |  | HMEL015425 | dual specificity protein kinase TTK-like |
| 95 | chr11 | HMEL015425 | dual specificity protein kinase TTK-like |
|  |  | HMEL015426 | spectrin alpha chain |
|  |  | HMEL015428 | protein vav-like |
| 96 | chr18 |  |  |
| 97 | chr18 |  |  |

**Table S9.** Autosome versus Z chromosome population genetic statistics.

1. ***D*-statistics** (all statistical tests are comparisons of the observed *D* value to the null hypothesis of zero).

|  | **autosome** | **Z** |
| --- | --- | --- |
| *D* (*besckei*, *ethilla*, *m. nanna*, *wallacei*) | 0.1667±0.0019*** | 0.4011±0.0203*** |
| *D* (*besckei*, *numata*, *m. nanna*, *wallacei*) | 0.1062±0.0015*** | 0.0362±0.0087*** |
| *D* (*numata*, *ethilla*, *m. nanna*, *wallacei*) | 0.0700±0.0020*** | 0.3942±0.0207*** |
| *D* (*ethilla*, *hecale*, *m. nanna*, *wallacei*) | 0.0692±0.0018*** | 0.0488±0.0140*** |
| *D* (*ethilla*, *pardalinus*, *m. nanna*, *wallacei*) | 0.0857±0.0017*** | 0.0897±0.0137*** |
| *D* (*hecale*, *pardalinus*, *m. nanna*, *wallacei*) | 0.0166±0.0013*** | 0.0377±0.0122** |
| *D* (*numata*, *pardalinu*s, *m. nanna*, *wallacei*) | 0.1495±0.0019*** | 0.4357±0.0195*** |
| *D* (*numata*, *pardalinus*, *m. rosina*, *wallacei*) | 0.1516±0.0018*** | 0.4380±0.0197*** |
| *D* (*numata*, *pardalinus*, *cydno*, *wallacei*) | 0.1082±0.0017*** | 0.4269±0.0199*** |

**indicates p<0.01

*** indicates p<0.001

1. **d_xy_**

|  | **autosome** | **Z** |
| --- | --- | --- |
| d_xy_ (*pardalinus*, *m. nanna*) | 0.0228±0.0001 | 0.0188±0.0004 |
| d_xy_ (*numata*, *m. nanna*) | 0.0240±0.0001 | 0.0235±0.0006 |
| d_xy_ (*pardalinus*, *m. rosina*) | 0.0215±0.0001 | 0.0178±0.0004 |
| d_xy_ (*numata*, *m. rosina*) | 0.0226±0.0001 | 0.0224±0.0006 |
| d_xy_ (*pardalinus*, *cydno*) | 0.0221±0.0001 | 0.0174±0.0004 |
| d_xy_ (*numata*, *cydno*) | 0.0229±0.0001 | 0.0221±0.0006 |

1. **F_ST_**

|  | **autosome** | **Z** |
| --- | --- | --- |
| F_ST_ (*pardalinus*, *m. nanna*) | 0.4525±0.0010 | 0.6391±0.0072 |
| F_ST_ (*numata*, *m. nanna*) | 0.6174±0.0010 | 0.7599±0.0048 |
| F_ST_ (*pardalinus*, *m. rosina*) | 0.2596±0.0015 | 0.5083±0.0094 |
| F_ST_ (*numata*, *m. rosina*) | 0.4238±0.0013 | 0.6482±0.0065 |
| F_ST_ (*pardalinus*, *cydno*) | 0.2242±0.0012 | 0.3674±0.0088 |
| F_ST_ (*numata*, *cydno*) | 0.3780±0.0010 | 0.5510±0.0047 |
